# Geoarchaeological evidence of the AD 1642 Yellow River flood that destroyed Kaifeng, a former capital of dynastic China

**DOI:** 10.1038/s41598-020-60169-1

**Published:** 2020-02-28

**Authors:** Michael Storozum, Peng Lu, Sanying Wang, Panpan Chen, Ruixia Yang, Qifeng Ge, Jinping Cao, Junwei Wan, Hui Wang, Zhen Qin, Haiwang Liu, Edward Park

**Affiliations:** 1 Institute of Archaeological Science, Fudan University, Shanghai, 200433 P.R. China; 2 Department of Cultural Heritage and Museology, Fudan University, Shanghai, 200433 P.R. China; 3 Department of Archaeology, Max Planck Institute for the Science of Human History, Jena, 07745 Germany; 4 Institute of Geography, Henan Academy of Sciences, Zhengzhou, 450052 P.R. China; 5 Kaifeng Institute of Archaeology and Cultural Relics, Kaifeng, 475000 P.R. China; 6 International Center on Space Technologies for Natural and Cultural Heritage Under the Auspices of UNESCO, Beijing, 100094 P.R. China; 7 Institute of Archaeology, Chinese Academy of Social Sciences, Beijing, 100710 P.R. China; 8 School of History and Culture, Henan University, Kaifeng, 475001 P.R. China; 9 Henan Provincial Institute of Cultural Relics and Archaeology, Zhengzhou, 450000 P.R. China; 10 National Institute of Education, Nanyang Technological University, Singapore, 639798 Singapore; 11 Asian School of the Environment, Nanyang Technological University, Singapore, 639798 Singapore

**Keywords:** Environmental social sciences, Natural hazards

## Abstract

Rising global temperatures will increase the number of extreme weather events, creating new challenges for cities around the world. Archaeological research on the destruction and subsequent reoccupation of ancient cities has the potential to reveal geological and social dynamics that have historically contributed to making urban settings resilient to these extreme weather events. Using a combination of archaeological and geological methods, we examine how extreme flood events at Kaifeng, a former capital of dynastic China, have shaped the city’s urban resilience. Specifically, we focus on an extreme Yellow River flood event in AD 1642 that historical records suggest killed around 300,000 people living in Kaifeng. Our recent archaeological excavations have discovered compelling geological and archaeological evidence that corroborates these documents, revealing that the AD 1642 Yellow River flood destroyed Kaifeng’s inner city, entombing the city and its inhabitants within meters of silt and clay. We argue that the AD 1642 flood was extraordinarily catastrophic because Kaifeng’s city walls only partly collapsed, entrapping most of the flood waters within the city. Both the geology of the Yellow River floods as well as the socio-political context of Kaifeng shaped the city’s resilience to extreme flood events.

## Introduction

As cities face growing environmental uncertainties brought on by human-induced climate change, scientists are becoming increasingly interested in the factors that make cities resilient to natural hazards^1–3^. Scientific studies on the sedimentary and archaeological records found underneath modern cities have the potential to reveal the geomorphic and social dynamics that have contributed to creating resilient urban environments^4,5^. In particular, archaeological layers found underneath modern cities near rivers can contain evidence of extreme flood events that are often outside the available range of instrumental records^6–8^. China’s Yellow River is a particularly fruitful context for this kind of research because of China’s long recorded history of Yellow River flood events and abundant archaeological remains. According to historical sources, the Yellow River has flooded over 1000 times in the past 2000 years, claiming millions of lives^9–11^. Recent archaeological and geological field work has found the physical evidence of several of these historically recorded flood events, but none of these studies have yet examined the influence catastrophic Yellow River flood events have had on shaping the resilience of large cities in the Yellow River’s floodplain^12–14^.

**Figure 1:**
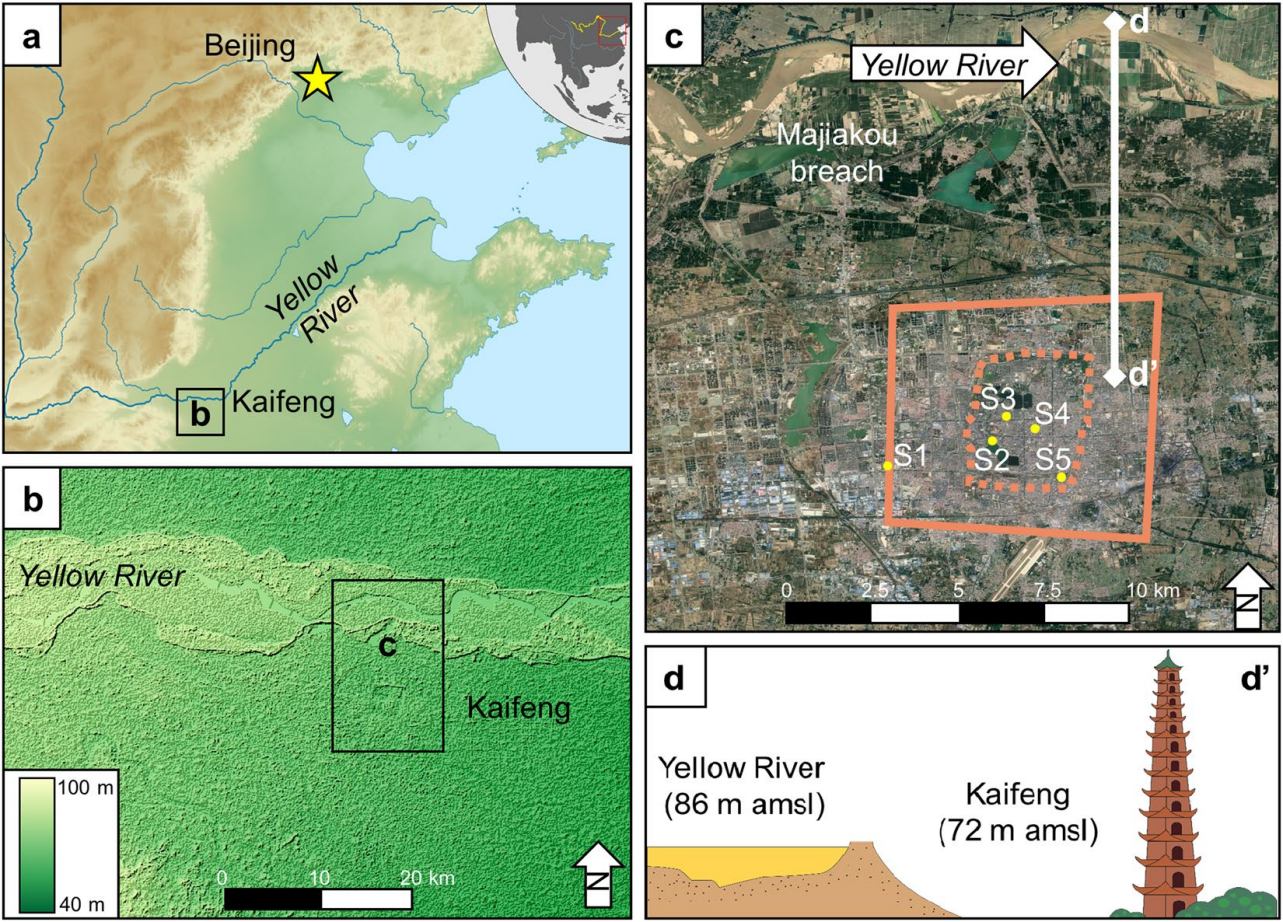
Kaifeng and the Yellow River. (**a**) Topography of the North China Plain. (**b**) SRTM 90 m DEM of the area around Kaifeng, note that the elevation of the Yellow River is higher than Kaifeng (Data available from the U.S. Geological Survey). (**d**) The city walls of Kaifeng (solid line is the Song dynasty wall, dashed line is the Ming /Qing dynasty wall) and sample locations Xinzhengmen (S1), Yongning Wangfu (S2), Dichen Xiyuan (S3), Xinjiekou (S4), and Yulongwan (S5) (Imagery from Google Earth Pro, Digital Globe). (**d**) The super elevated channel of the Yellow River hanging above Kaifeng, with the tallest building in medieval Kaifeng, the Iron Pagoda, for scale.

Kaifeng was one of the largest cities in the world, the imperial capital of several Chinese dynasties, and also an epicenter for Yellow River flood events (Fig. 1a–d). Despite Kaifeng’s political and economic importance, the Yellow River has flooded Kaifeng around 40 times over the past 3000 years. These frequent floods have left behind meters of alluvium mixed with urban debris, creating a 20 m thick archaeological sequence that dates from the Bronze Age (ca. 2000 BC) to the Qing dynasty (AD 1644–1912) (Supplementary Table 1). However, only one of these floods was powerful enough to destroy the entire city. According to *The Veritable Documents of the Ming*, in AD 1642 a large army breached the dikes along the Yellow River to intentionally flood the city, killing nearly 300,000 people^15^. Kaifeng’s archaeological record contains ample evidence of urban destruction and renewal as a result of this flood event and many others, making it an ideal location to start to examine the geological and social components of urban resilience in relation to the Yellow River^16–18^.

Recent archaeological work at Kaifeng has unearthed compelling archaeological and sedimentary evidence that corroborates the historical record of the exceptionally destructive AD 1642 Yellow River flood and other historically documented flood events. In this paper, we present archaeological evidence, radiocarbon dates, and geological data from excavations at excavations at Xinzhengmen, one of the western gates of the Song dynasty (AD 960-1128) city, and several Ming dynasty (AD 1368-1642) “princely houses” known as *Wangfu*, to provide insights into the social and natural factors that contributed to both the destruction and subsequent recovery of Kaifeng after this devastating flood event.

## Results

Since the 1980s’, the Kaifeng Institute of Archaeology and Cultural Relics has conducted numerous salvage excavations throughout the municipal area of Kaifeng (see Supplementary Information). We have gathered stratigraphic, radiocarbon, and archaeological evidence that strongly supports the historical descriptions of the AD 1642 Yellow River flood from five recently excavated locations: Xinzhengmen, Yongning Wangfu, Dichen Xiyuan, Xinjiekou, and Yulongwan (see Fig. 1c, Table 1, and Supplementary Table 2).

**Table 1:** Accelerator mass spectrometry radiocarbon dates from Xinzhengmen, Yongning Wangfu, Yulongwan, and Dichen Xiyuan. Dates for each site are presented from oldest to youngest. The * indicates the date is anomalous. Dates are calibrated with BetaCal 3.21 using the IntCal13 dataset.

Site and layer	Material	Lab #	Context	^14^C years	*δ*^13^C	Calibrated years AD (2σ)	Calibrated years BP (2σ)	Probability (%)
Xinzhengmen* (S1-7)	Organic sediment	Beta 535782	Context G3, flood channel, 67 m amsl	7120 ± 30	−24.8	6057-5977 BC 5947-5921 BC	8006-7926 7896-7870	83.4 12.0
Xinzhengmen (S1-8)	Charcoal	Beta 535781	Context L14, 66.1 m amsl	950 ± 30	−24.5	1024-1155	926-795	95.4
Xinzhengmen (S1-6)	Charcoal	Beta 535783	Context H231, 68.5 m amsl	220 ± 30	−25.1	1735-1806 1642-1684 1933-post 1950	215-144 308-266 17 – post 0 BP	46.2 37.9 11.3
Xinzhengmen (S1-3)	Wood	Beta 500051	Buried tree, bark, found within AD 1841 flood deposit	130 ± 30	−21.8	1798-1894 1674-1778 1905-1942	152-56 276-172 45-8	42.4 38 14.9
Yongning Wangfu (S2-4)	Tooth collagen	Beta 535791	T15, F9, #86	390 ± 30	−17.6	1440-1524 1571-1630 1559-1562	510-426 379-320 391-388	69.2 25.7 0.5
Yongning Wangfu (S2-4)	Tooth collagen	Beta 535792	T15:2, F9, #92	330 ± 30	−18.1	1477-1642	473-308	95.4
Yongning Wangfu (S2-4)	Wood	Beta 500052	Buried tree, bark, found within AD 1642 flood deposit	340 ± 30	−23.9	1470-1640	480-310	95.4
Dichen Xiyuan (S3-2)	Wood	Beta 535785	Buried tree, bark, found within AD 1642 flood deposit	280 ± 30	−21.9	1512-1600 1616-1666 1784-1795 1498-1502	438-350 334-284 166-155 452-448	54.3 38.3 2.1 0.6
Yulongwan (S5-3)	Tooth collagen	Beta 535789	T1:2, *Jian* 6	340 ± 30	−15.2	1470-1640	480-310	95.4
Yulongwan (S5-3)	Tooth collagen	Beta 535790	T2, F1, *Jian* 11	330 ± 30	−13.8	1477-1642	473-308	95.4

### Xinzhengmen (S1)

**Figure 2:**
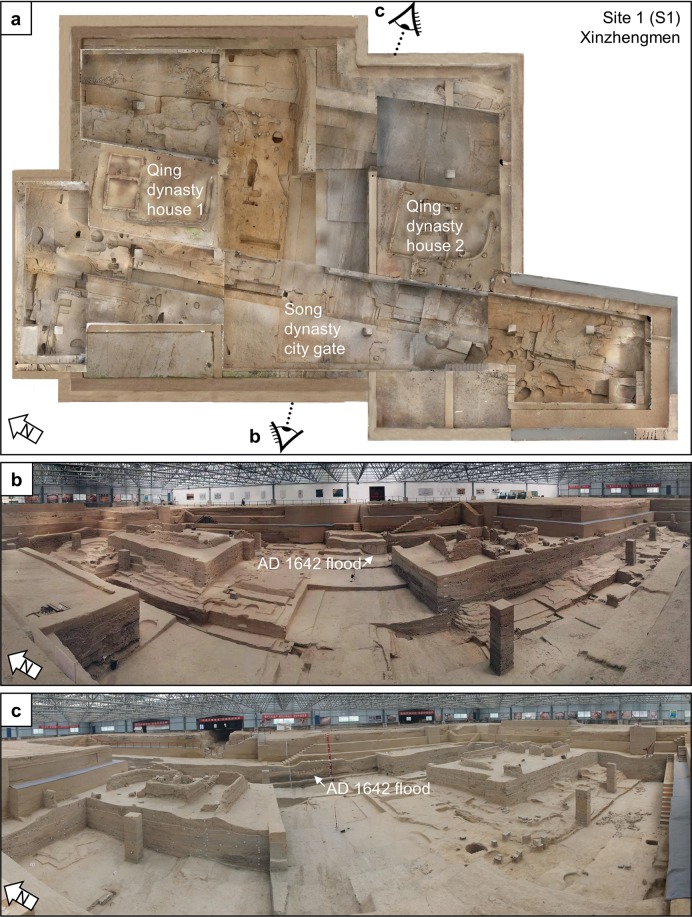
Xinzhengmen. (**a**) Plan view of of Xinzhenmen with panoramas taken from locations (**b**,**c**). (**b**) View to the east. (**c**) View to the west.

At Xinzhengmen, archaeologists have opened up a 2,100 m^2^ excavation area, uncovering many architectural features that date to the Song, Yuan, Ming, and Qing dynasties^18^ (Fig. 2a). Two sections, one in the north and one in the south, were excavated down to the Qing dynasty land surface, while the rest of the excavation exposes the Song dynasty land surface. The trench is approximately 8 m deep and contains the remains of the Song dynasty city wall and gate (Fig. 2b). The foundations of the Song dynasty city wall are 23 m wide and are composed of rammed earth. The northern and southern sections of the excavation are about 4 m deep and contain evidence of several Qing dynasty house compounds buried by a Yellow River flood in AD 1841. Most of the exposed stratigraphy dates to the Ming dynasty occupation of the city, which itself contains a flood deposit that incised through many Ming dynasty strata (Fig. 2c). According to the ceramics found within these strata, archaeologists date this incised flood deposit to the AD 1642 Yellow River flood event.

**Figure 3:**
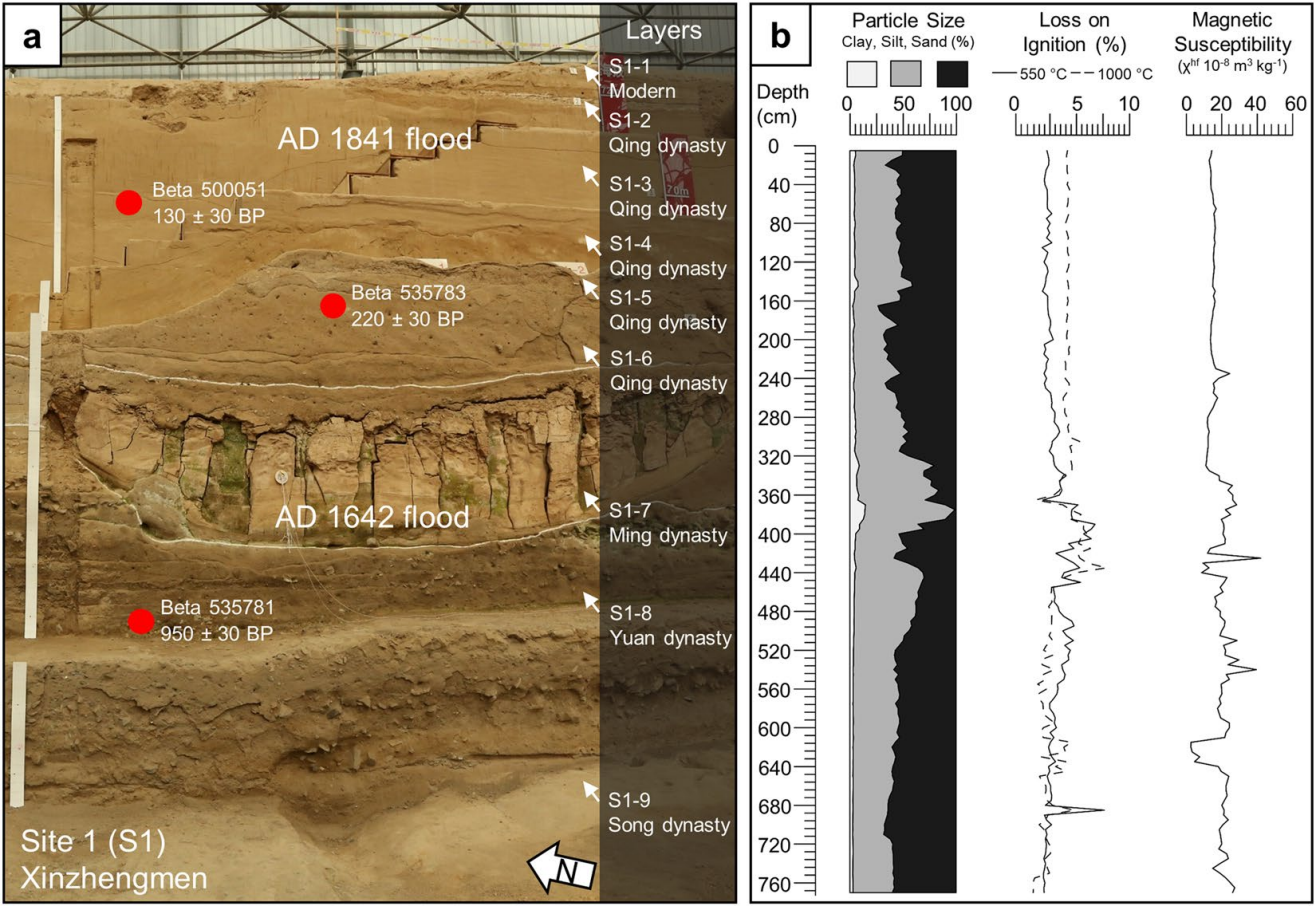
Stratigraphy at Xinzhengmen. (**a**) Sedimentary sequence including the incised deposit of the AD 1642 Yellow River flood and the overlying AD 1841 Yellow River flood, the red circles are placed at the approximate depths of samples for radiocarbon dating, see Supplementary Information for more details (**b**), data for particle size, loss on ignition, and magnetic susceptibility every 5 cm.

We collected three radiocarbon samples from the eastern profile to determine the age of these flood deposits (Fig. 2c, Supplementary Fig. 1, and Table 1). We constrained the chronology of the incised channel by collecting two samples, one from below and one from above the incised channel, to provide a *terminus post quem* and a *terminus ante quem* for the flood deposit (Fig. 3a). The sample (Beta 53781) located directly beneath the incised flood channel returned a date of 950 ± 30 BP, which calibrated to AD 1024-1155 (95.4%) (Supplementary Fig. 2a). The second sample, 1.5 m above the incised flood deposit (Beta 535783), returned a date of 220 ± 30 BP, which calibrated to AD 1735-1806 (46.2%), AD 1642-1854 (37.9%), and AD 1933-post 1950 (11.3%) (Supplementary Fig. 2b,3a). Subsequent calibration of the first pair of dates at Xinzhengmen constrained the age of the flood deposit, making it highly likely that this incised flood deposit is part of the AD 1642 Yellow River flood event. A third sample (Beta 500051) from Xinzhengmen was collected from a buried tree found within the AD 1841 flood deposit and returned at date of 130 ± 30 BP, which calibrates to AD 1798-1894 (42.4%), AD 1674-1778 (38.0%), AD 1905-1942 (14.9%) (Supplementary Fig. 3b).

The excavations at Xinzhengmen have revealed a near continuous record of sedimentation from the Song to the Qing dynasties (Fig. 3a,b). Based on the radiocarbon and ceramic evidence, the stratigraphy contains deposits from the historically recorded Yellow River floods of AD 1642 and AD 1841. According to the particle size data collected from the eastern profile, the sediments from the Song dynasty and the Qing dynasty occupation layers are sandy loams that have higher magnetic susceptibilities than both the AD 1642 and AD 1841 Yellow River flood deposits (Fig. 3b). The AD 1642 Yellow River flood deposit is composed of two parts, a reddish silt-filled channel and an underlying sandy layer, presumably the channel’s bedload. Both parts of the AD 1642 flood deposit cleanly incised through cultural deposits, including cobble roads, bricks, and pottery, which provides a relative sense for the power of this flood event (Supplementary Fig. 4a,b). Around 2 m above the AD 1642 incised flood deposit is a 3 m thick sandy loam deposit that corresponds to the AD 1841 Yellow River flood. This flood deposit cleanly overlies the early Qing dynasty archaeological deposits at Xinzhengmen. This flood deposit contain many laminated beds that likely represent episodic flooding that occurred over many months, corroborating descriptions of the AD 1841 flood found in historical documents^19,20^.

### Yongning Wangfu (S2)

**Figure 4:**
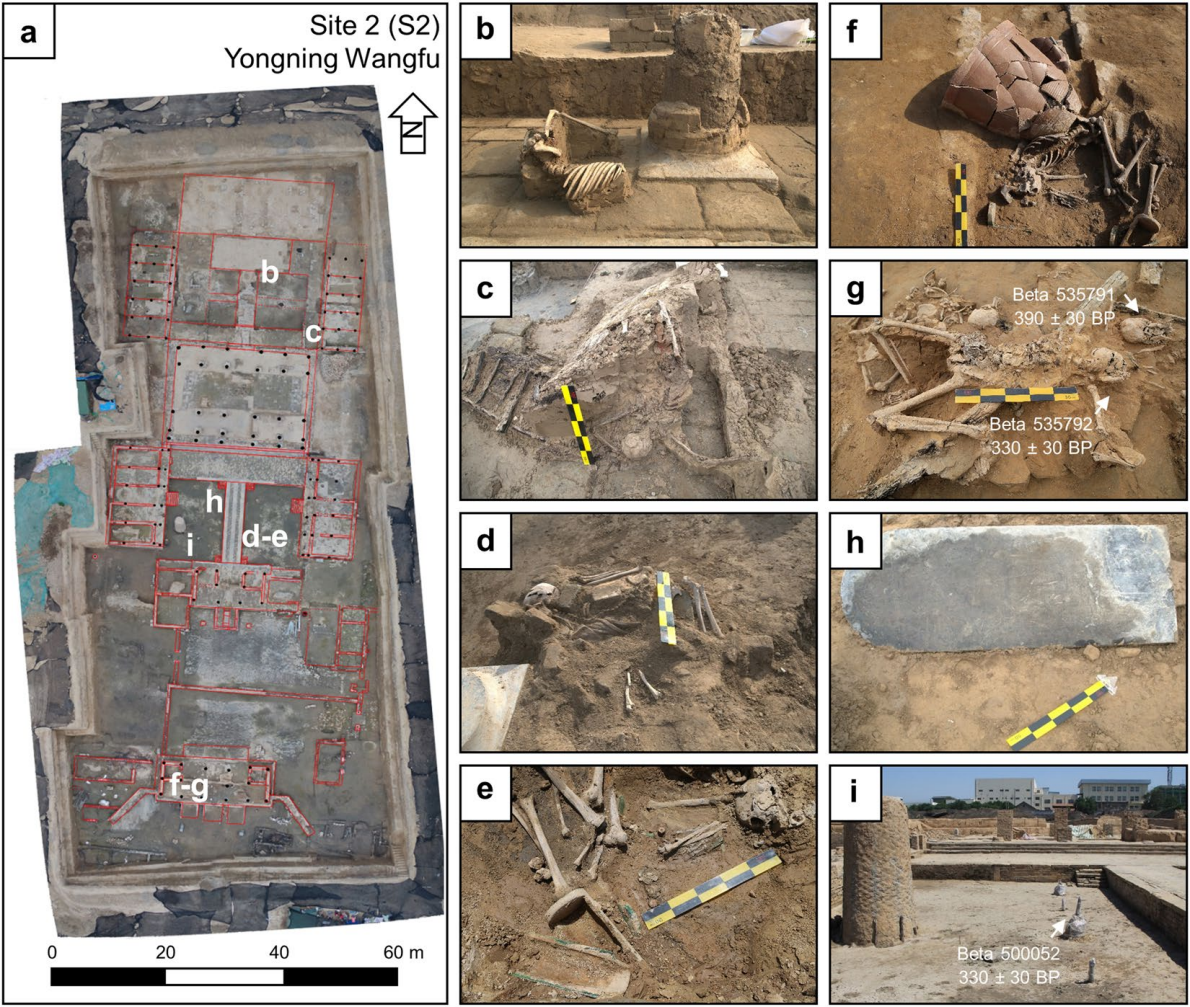
Yongning Wangfu. (**a**) Plan view of excavations at Yongning Wangfu with locations of AD 1642 flood victims and chronological information marked. (**b–g**) Victims of the AD 1642 flood. (**h**) Stelae dating to AD 1695 found in reoccupation layer. (**i**) Wood column sampled for radiocarbon dating.

At Yongning Wangfu, archaeologists excavated around 4,000 m^2^ to reveal the layout of a palatal compound where they found over 1,000 sets of porcelain and copperware, as well as stone tablets inscribed with family lineages and reign dates (Fig. 4a, Supplementary FigS. 5–7). Here, archaeologists have also found 15 skeletonized individuals that perished before or during the AD 1642 flood (Fig. 4b–g). Most of the human remains are highly fragmentary and disarticulated. The individuals that were articulated or partially articulated were found in a variety of positions, including both prone and supine with flexed limbs. Two individuals were found within wooden coffins and are presumed to have deceased before the AD 1642 flood. Aside from the human remains found within coffins, all the other skeletonized individuals appear to have died violently in the flood, disarticulated by debris and then buried within the mud left behind from the flood waters. These individuals are overlain by debris found within the flood deposit, including a wooden beam that appears to have penetrated an individual’s cranium and a large clay vessel which intrudes into another individual’s abdomen (Fig. 4f,g). Several meters above the AD 1642 flood deposit, archaeologists found a stela inscribed with the date of the 34^th^ year of the Kangxi emperors’ reign, or AD 1695, indicating that people reoccupied the city around 50 years after the AD 1642 flood (Fig. 4h, see Supplementary Fig. 8b).

At Yongning Wangfu, we have obtained three radiocarbon dates, one of which comes from a wooden column and the other two dates are from skeletonized individuals disarticulated by flood debris (Supplementary Fig. 9a,b, Table 1). One individual (Beta 535791) returned a radiocarbon date of 390 ± 30 BP, calibrated to 1440–1524 (69.2%), 1571–1630 (25.7%), 1559–1562 (0.5%), roughly corresponding with the AD 1642 flood event and the other individual (Beta 535792) returned a radiocarbon age of 330 ± 30 BP, calibrated to 1477–1642 (95.4%) (Fig. 4g). The wood sample (Beta 500052) returned a date of 340 ± 30 BP, calibrated to AD 1470–1640 (95.4%) (Fig. 4i).

**Figure 5:**
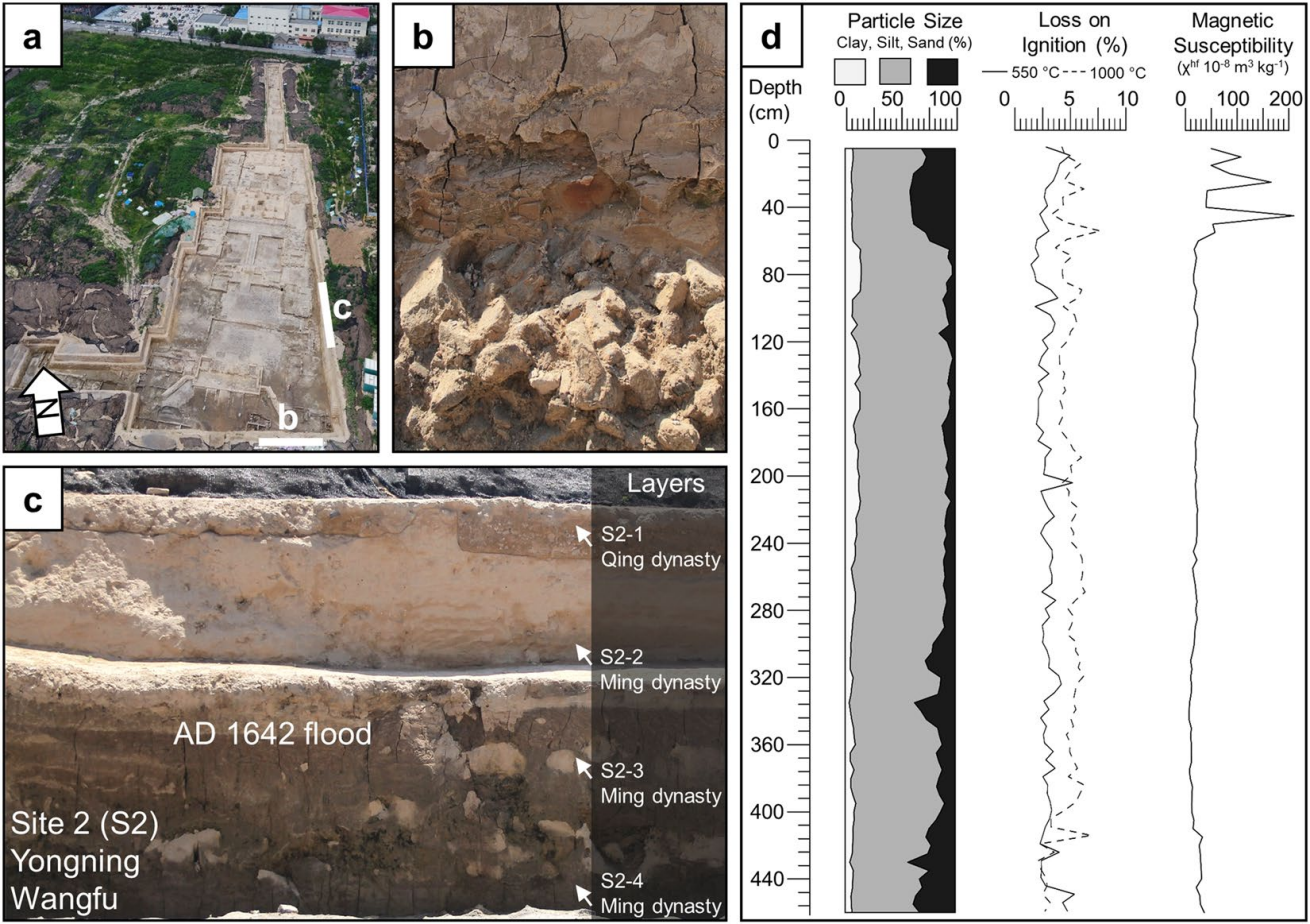
Stratigraphy at Yongning Wangfu. (**a**) Plan view of Yongning Wangfu with locations of profiles marked. (**b**) Southern profile with brick debris covered by silty clay loam with redoximorphic features. (**c**) eastern profile with AD 1642 flood deposit and overlying Qing dynasty reoccupation. (**d**) Data for particle size, loss on ignition, and magnetic susceptibility every 5 cm.

The stratigraphy at Yongning Wangfu reveals that the AD 1642 Yellow River flood deposit is approximately 3 m thick and is primarily composed of silt loam (Fig. 5a–c, Supplementary Fig. 10). This profile contains stratigraphic evidence of the destructive power of the AD 1642 Yellow River flood as well as cultural layers that date to the reoccupation of the city during the early Qing dynasty. Large silt and sand “rip-up” clasts show that the flood was extremely turbid and that the mudbrick buildings dissolved into the otherwise clayey matrix of the Yellow River flood deposit (Fig. 5c). According to our particle size data, the flood deposit becomes less sandy up column, probably a result of the general reduction of the flood’s energy (Fig. 5d). As the flood lost energy, it deposited a reddish silt loam, typical of Yellow River flood deposits found nearby^14,21^. The flood deposits are clearly seen in the profiles exposed during the excavations at Yongning Wangfu. The high magnetic susceptibility values found in the Qing dynasty layer corroborates the archaeological evidence for a rapid reoccupation of Kaifeng after its destruction in AD 1642.

### Dichen Xiyuan (S3)

**Figure 6:**
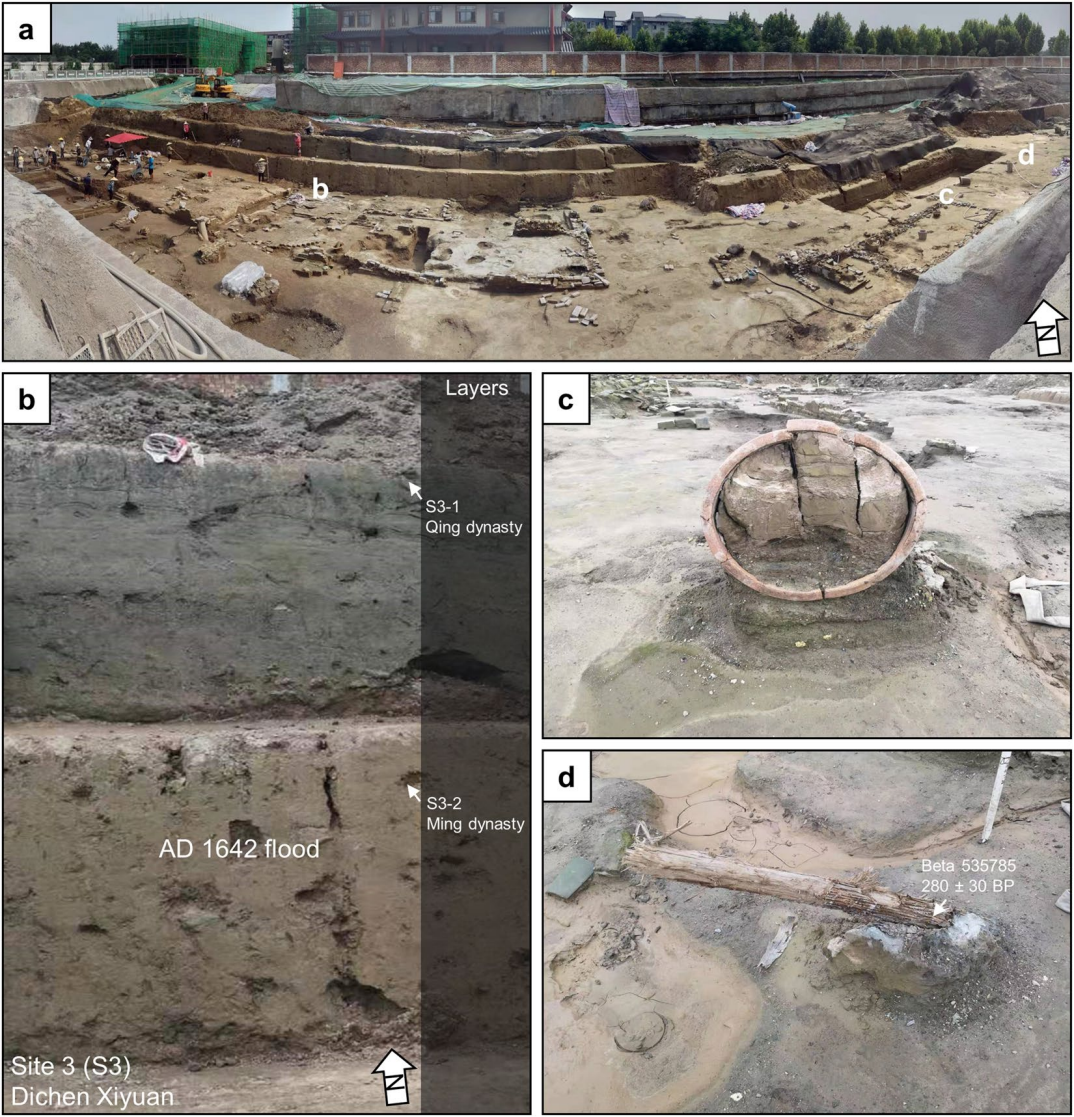
Dichen Xiyuan. (**a**) Panorama of excavations at Dichen Xiyuan. (**b**) Intact stratigraphic section of the AD 1642 Yellow River flood. (**c**) Overturned ceramic vessel containing intact AD 1642 flood stratigraphy. (**c**) Buried tree sampled for radiocarbon dating.

At Dichen Xiyuan, archaeologists opened up a 3,700 m^2^ rescue excavation. Here, archaeologists found a large concentration of Ming dynasty buildings with pottery, porcelain and the remains of a large kiln (Fig. 6a–c, Supplementary Fig. 11a–c). Judging from the layout of the buildings, the site may be the western part of the *Zhou Wangfu*. We collected a piece of bark from a buried tree in a courtyard of a building for radiocarbon dating and the sample (Beta 535785) returned a date of 280 ± 30 BP, which calibrates to AD 1512–1600 (54.3%), AD 1616–1666 (38.3%), AD 1784–1795 (2.1%), and AD 1498–1502 (0.6%) (Fig. 6d, Table 1).

### Xinjiekou (S4)

**Figure 7:**
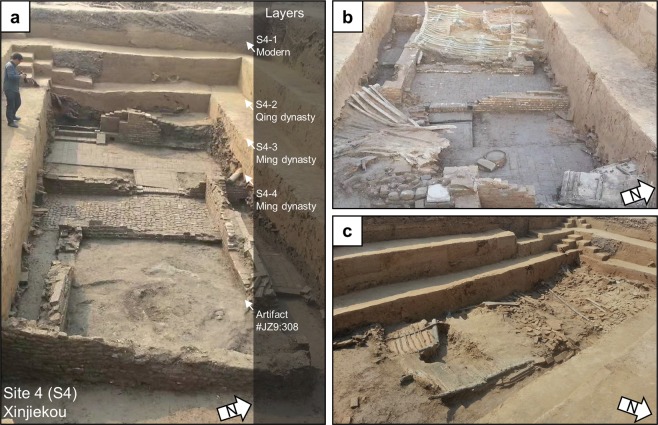
Xinjiekou. (**a–c**) Excavations at Xinjiekou where the wooden plaque was found (artifact number JZ9: 308).

At Xinjiekou, archaeologists conducted rescue excavations and uncovered over 400 m^2^ of the former location of the *Dianyi Suo* (Office of the Rites), which was destroyed in the AD 1642 flood^22^. The archaeologists found artifacts that date to the late Ming through the late Qing dynasties, including a Ming dynasty courtyard that had collapsed houses and walls (Fig. 7a–c). Many artifacts were excavated from the site, including lacquered wooden wares, porcelain, pottery and other daily utensils. Within the courtyard, archaeologists unearthed a wooden plaque inscribed with the date of the tenth year of Emperor Chongzhen’s reign (AD 1637), providing a reliable chronological anchor for the site (see Supplementary Information).

### Yulongwan (S5)

**Figure 8:**
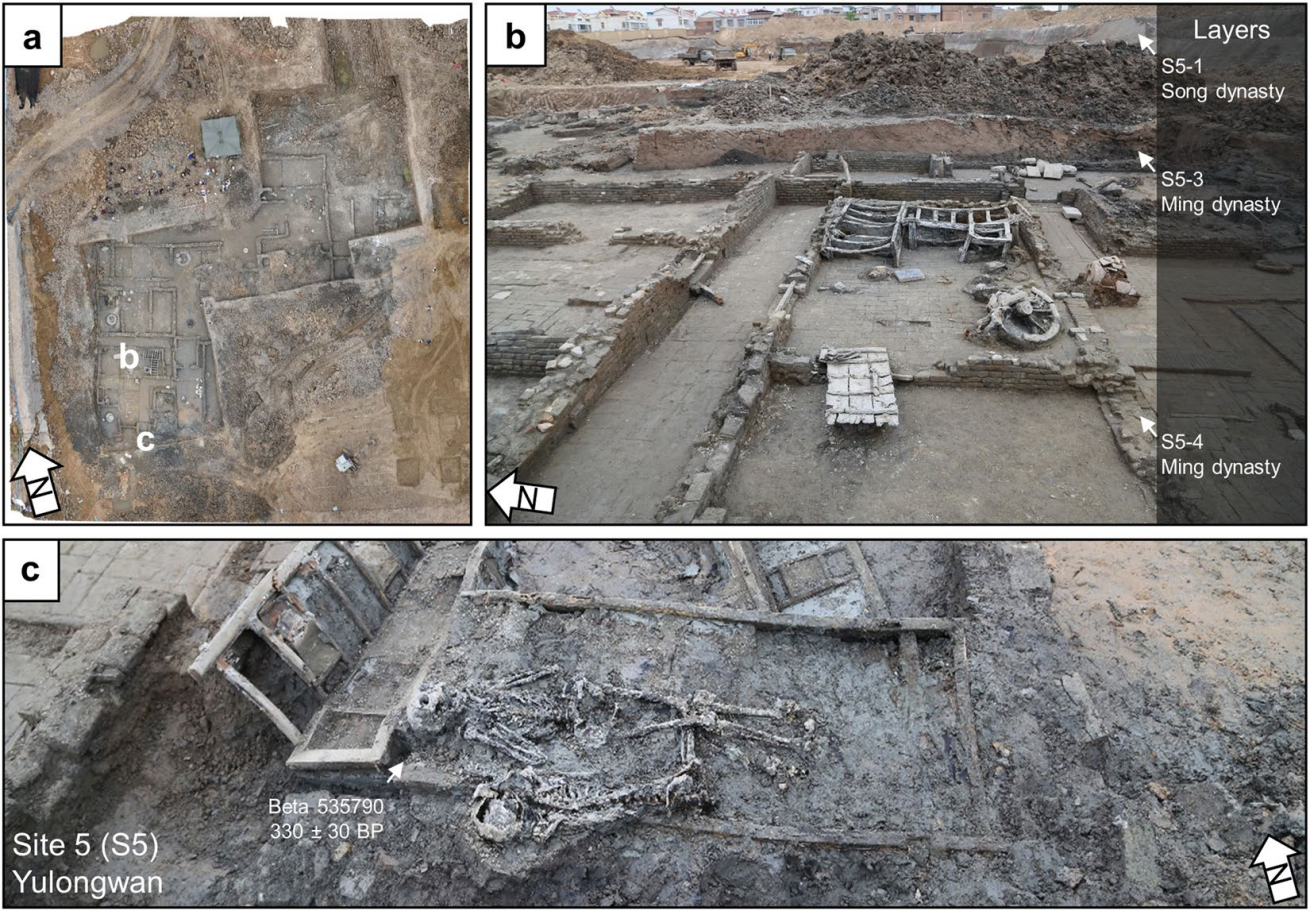
Yulongwan. (**a**) Plan view of Yulongwan with locations of buildings and flood victims marked. (**b**) Building with remains of wooden wheels and doors. (**c**) Two individuals found prone on a wooden bed.

At Yulongwan, archaeologists opened up an area of 970 m^2^ as part of a rescue excavation (Fig. 8a). The archaeologists found three buildings that date to the late Ming dynasty which contained over 3,000 artifacts (Fig. 8b). Many of these artifacts are coins that were cast during the late Ming dynasty. Archaeologists also found 11 skeletonized individuals, four of which were badly disarticulated, likely a result of a violent death in the AD 1642 flood. Among these individuals, two were found prone on a wooden bedframe (Fig. 8c). We sampled two individuals found at the site for radiocarbon dating (Table 1). One individual (Beta 535789) returned an age of 340 ± 30 BP, which calibrates to AD 1470-1640 (95.4%), and another (Beta 535790) returned a radiocarbon age of 330 ± 330 BP, which calibrates to AD 1477-1642 (95.4%), supporting the chronology provided by the material evidence found at the site (Fig. 8c).

## Discussion

**Figure 9:**
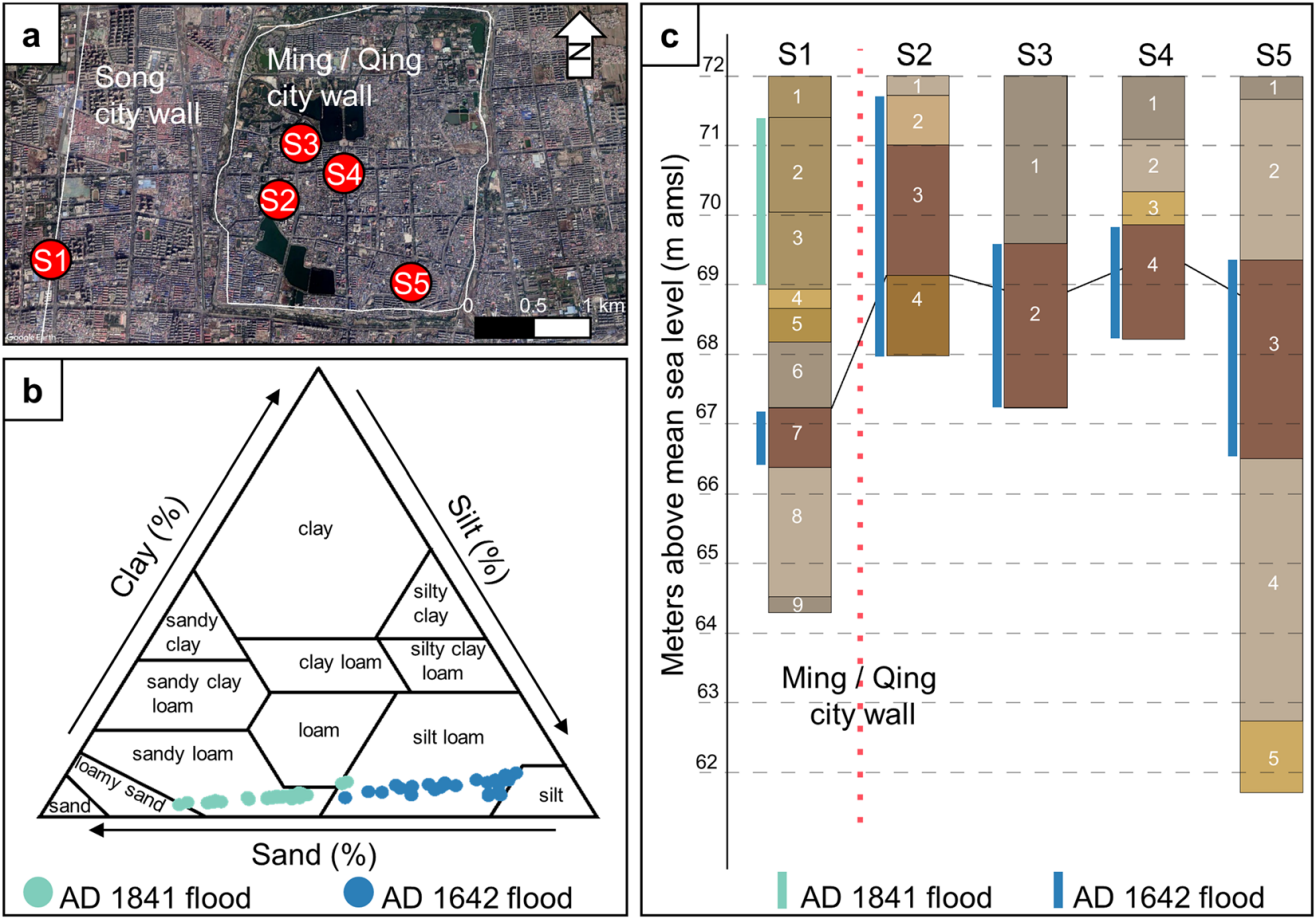
Urban stratigraphy of Kaifeng. (**a**) Locations of sites mentioned in the text, Xinzhengmen (S1), Yongning Wangfu (S2), Dichen Xiyuan (S3), Xinjiekou (S4), and Yulongwan (S5) (Imagery from Google Earth Pro, Digital Globe). (**b**) Particle size differences between the AD 1642 and AD 1841 Yellow River flood deposits. (**c**) Summarized stratigraphic evidence from each excavation, the black line connects each of the AD 1642 flood deposits. Note that the flood deposit is found several meters deeper beneath the modern land surface outside the Ming dynasty city walls than inside the city walls.

Recent excavations of Kaifeng’s archaeological record are already revealing a unique dataset that is useful for understanding the many geological and social factors that compose urban resilience in relation to the Yellow River flood events. Taken together, the archaeological, historical, and sedimentary data recovered from Kaifeng provide enough information to tentatively reconstruct the fluvial dynamics of the AD 1642 flood using paleohydraulic methods (Fig. 9a). According to the historical records, the levee was breached somewhere around modern-day Majiakou village, about 10 km to the north of Kaifeng^15^. After the levee was breached, we estimate that the river’s water level dropped somewhere between 10 m to 15 m. We argue that these dropdown rates are accurate given that the Yellow River’s average depth is around 35 m near Kaifeng, which we estimated from the width to depth ratio of anabranching rivers found in climatic conditions similar to the Yellow River. Using Walder & O’Connor’s hydraulic equation to estimate the coefficients necessary to model the peak discharge of flash flood events as function of the drop in water level, we calculated that the flood’s peak discharge would be 360 m^3^ s^−1^ or 725 m^3^ s^−1^, given a 10 m or 15 m of drop in water level, respectively^23^.

Depending on the drop in water level, our values account for 32% or 64% of the river’s total discharge, meaning that a significant percentage of the Yellow River’s total discharge was diverted directly into the city almost immediately after the levee breach. Assuming the length of the breached dike is around 20 m, our rough estimate for the river’s velocity during peak discharge is around 3.6 m s^−1^ or 5.9 m s^−1^, a comparatively fast flow for a river in a lowland environment. This high flow velocity enabled the Yellow River to entrain objects as large as a meter in diameter, assuming a 15 m drop in water level^24^. The fast moving Yellow River, and the large objects the flood carried, could effectively scour through Kaifeng’s protective walls and gates, enabling the floodwaters to enter the city.

Archaeologists have found the most debris, rip-up clasts, and flood victims within the walled portion of Ming dynasty Kaifeng, supporting this paleohydraulic scenario. The flood deposits at Yongning Wangfu are more silt rich than the deposits outside the city wall, likely due to the fluvial dynamics of the AD 1642 flood that predominately affected the area within the Ming dynasty city walls (Fig. 9b). Inside the city walls, the AD 1642 Yellow River flood deposits are about 2 m beneath the modern ground surface and contain no clear stratigraphic evidence of the AD 1841 Yellow River flood. Outside the Ming dynasty city wall at Xinzhengmen, around 5 m of AD 1841 Yellow River flood sediment overlies the AD 1642 flood deposits. Based on this stratigraphic evidence, it is clear that in AD 1642, at least part of Kaifeng’s city walls collapsed, directing most of the floodwater straight into the inner city (Fig. 9c).

**Figure 10:**
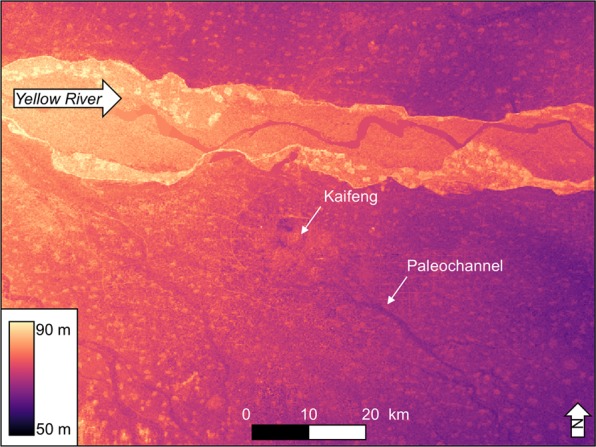
90 m SRTM DEM of Kaifeng and surrounding area. The DEM highlights the paleochannels around the site, specifically, the paleochannel coming out to the southeast of Kaifeng may be the relict channel of the AD 1642 flood deposit (Data available from the U.S. Geological Survey).

In central China, people built city walls to protect their cities against invading armies and extreme flood events^25^. In AD 1841, an extreme Yellow River flood inundated the area around Kaifeng, but the sedimentary evidence suggests that Kaifeng’s city walls successfully prevented most, if not all, of the flood waters from entering the inner city. However, in AD 1642, Kaifeng’s city walls collapsed under siege and subsequent scouring action from the Yellow River. Once breached, Kaifeng’s city walls provided no protection from the flood, and may have actually prevented the floodwaters from quickly exiting the inner city. As a result, Kaifeng’s city walls unexpectedly amplified the destructive power of the AD 1642 Yellow River flood. Eventually, the floodwaters exited the walled city to the southeast, leaving behind an incised channel seen in modern topography (Fig. 10). In combination, these multiple lines of evidence suggest that Kaifeng’s resilience to Yellow River flood events depended on the magnitude and duration of the floods as well as the social and political factors that determined the urban layout of the city.

## Conclusion

In this paper, we have presented compelling archaeological, radiocarbon, and sedimentary evidence of the historically documented Yellow River floods of AD 1642 and AD 1841 at Kaifeng. Our results corroborate historical accounts of the AD 1642 Yellow River flood that killed around 300,000 people and nearly destroyed Kaifeng, and the AD 1841 Yellow River flood that inundated the countryside. Using geoarchaeological and paleohydraulic methods, our tentative reconstruction of these Yellow River flood events indicates that, under different circumstances, Kaifeng’s city walls minimized or amplified the destructive potential of extreme Yellow River floods. Under normal circumstances, Kaifeng’s city walls prevented floodwaters from entering the city, but if the floodwaters breached the city walls, which happened during the AD 1642 flood, the city walls prevented water from easily exiting the city. As a result, the constant influx of floodwater into the city created a deadly mix of mud and urban debris that significantly amplified the destructive power of the Yellow River.

Our geoarchaeological investigations at Kaifeng suggest that urban resilience is not static but instead varies depending on the magnitude and type of natural hazard, the built landscape, as well as the city’s social institutions. As global temperatures continue to rise and increase the frequency of extreme events, the combined archaeological and paleoenvironmental record of exceptional floods, like the AD 1642 Yellow River flood, can provide an important reminder that unexpected events have happened in the past and will likely happen again. In extreme cases, these events can cause infrastructure built to prevent disasters to catastrophically fail, causing significantly more devastation than under normal circumstances.

## Methods

No statistical methods were used to predetermine sample size.

### Soil profiles and samples from excavation

The results presented here are derived from salvage field work conditions at Xinzhengmen, Yongning Wangfu, Dichen Xiyuan, Xinjiekou, and Yulongwan, limiting our ability to present accurate geodetic information and artifact distribution data. Samples and measurements of the lithostratigraphy and archaeostratigraphy at each site use standardized soil science terminology^26^. All bulk samples were collected at 5 cm intervals up column.

### Sediment analysis

We sampled each stratigraphic layer identified at Yongning Wangfu and Xinzhengmen and conducted particle size analysis, loss-on-ignition, and magnetic susceptibility on each sample. Particle size analysis was done using a Malvern 2000 particle size analyzer. We conducted loss on ignition at 550 °C and 950 °C to estimate the percentage of organic matter and inorganic carbon in each sample^27,28^. We also used a Bartington MS2 Magnetic Susceptibility Meter to determine each sample’s high field magnetism (4.7 kHz)^29^.

### Radiocarbon dating

We collected nine radiocarbon samples from wood, charcoal, and human remains and sent them for accelerator mass spectrometry radiocarbon dating at Beta Analytic. Accelerator mass spectrometry radiocarbon dates from Xinzhengmen, Yongning Wangfu, Yulongwan, and Dichen Xiyuan. Dates are calibrated with BetaCal 3.21 using the IntCal13 dataset^30,31^.

### Paleohydrology

Using the bulk density of typical clay material, (1.6 g/cm^3^) we calculated that about 6.4 tons of clay are needed to deposit 4 m of sediment over 1 m^2^ area. This means that at least 6.4 m of water column stacked over 1 m^2^ of water is necessary (i.e. 6.4 m^3^ of volume) in a relatively stagnant condition so that sediments could settle down, assuming the flooded water’s suspended sediment concentration was 1,000 mg/l.

## Supplementary information

Supplementary Information.

Supplementary Information 2.

## Data Availability

All data generated or analyzed in this study are included in the paper and its Supplementary Information. Site descriptions, radiocarbon dates, and ternary plots of particle size are presented in Supplementary Information. All remaining soil samples are curated at the Kaifeng Institute of Archaeology and Cultural Relics.
