# Supplementary material for: Geoarchaeological evidence of the AD 1642 Yellow River flood that destroyed Kaifeng, a former capital of dynastic China
Source: Sci Rep. 2020 Feb 28;10:3765. doi: 10.1038/s41598-020-60169-1 (PMC7048742; doi:10.1038/s41598-020-60169-1)
Supplement: Supplementary file 1 — Supplementary Information. [file 41598_2020_60169_MOESM1_ESM.pdf]

Geoarchaeological evidence of the AD 1642 Yellow River flood that destroyed Kaifeng, a former capital of dynastic China

Michael Storozum[1,2,3]*, Peng Lu[4]*, Sanying Wang[5]*, Panpan Chen[4], Ruixia Yang[6], Qifeng Ge[5], Jinping Cao[5], Junwei Wan[5], Hui Wang[7], Zhen Qin[8], Haiwang Liu[9], Edward Park[10,11]

1. Institute of Archaeological Science, Fudan University, 220 Handan Road, Shanghai, P.R. China
2. Department of Cultural Heritage and Museology, Fudan University, Shanghai, P.R. China
3. Max Planck Institute for the Science of Human History, Jena, Germany
4. Institute of Geography, Henan Academy of Sciences, Zhengzhou 450052, P.R. China
5. Kaifeng Institute of Archaeology and Cultural Relics, Kaifeng 475000, P.R. China
6. International center on space technologies for natural and cultural heritage under the Auspices of UNESCO, Beijing 100094, P.R. China
7. Institute of Archaeology, Chinese Academy of Social Sciences, Beijing 100710, P.R. China
8. School of History and Culture, Henan University, Kaifeng 475000, P.R. China
9. Henan Provincial Institute of Cultural Relics and Archaeology Zhengzhou 450000, P.R. China
10. National Institute of Education, Nanyang Technological University, Singapore, Singapore
11. Asian School of the Environment, Nanyang Technological University, Singapore, Singapore

*Correspondence to: mjstorozum@fudan.edu.cn, bulate_0@163.com, 125615966@qq.com

This file includes:

1. Kaifeng's geography and history
2. Textual records
   - 2.1 The AD 1642 Yellow River flood
   - 2.2 The AD 1841 Yellow River flood
3. Excavated sites at Kaifeng
   - 3.1 Xinzhengmen (S1)
   - 3.2 Yongning Wangfu (S2)
   - 3.3 Dichen Xiyuan (S3)
   - 3.4 Xinjiekou (S4)
   - 3.5 Yulongwan (S5)
4. Supplementary References
5. Supplementary Figures 1-11
6. Supplementary Tables 1 and 2

1. Kaifeng's geography and history

Situated in the middle of the North China Plain, Kaifeng was a geographic pivot of ancient and medieval China[1]. If the emperor controlled Kaifeng and the roads and waterways that ran through it, he controlled the northern and southern reaches of his empire. However, the superior geo-political positioning of Kaifeng came at a cost – the main course of the Yellow River is only 10 km to the north of the city and required a constantly vigilant and competent class of bureaucrat-engineers to maintain the levees that impounded and constrained the course of the Yellow River. Since the Bronze Age, emperors and kings of Kaifeng have tried to manage and mitigate the Yellow River's flood risk[2]. Nonetheless, despite their precautions the river still catastrophically breached its banks multiple times throughout history, burying the city under meters of alluvium.

Unlike many other river cities, a topographic quirk particular to the Yellow River poses an existential threat to the city of Kaifeng. The Yellow River's middle course flows through the easily erodible Loess Plateau where it accumulates hundreds of millions of tons of sediment before it reaches the North China Plain[3]. As the slope of the Yellow River flattens out after the river reaches its lower course near Sanmenxia, the sediment entrained in river's middle reaches starts to fall out of suspension, gradually elevating the main channel of the Yellow River by centimeters a year[4]. Over the course of several thousand years, the accumulation of silt elevated the Yellow River's main channel by about 10 m above the land surface of Kaifeng[5]. The elevated channel of the Yellow River means that a wall of water will come spilling out if the levees impounding the river are breached.

Despite the extraordinary risk of Yellow River flooding, Kaifeng's geo-politically advantageous position has helped sustain its centrality for thousands of years[6]. Although the name of the city has changed many times over the millennia, the city's location has essentially remained the same (Supplementary Table 1). The first well-documented city at Kaifeng is the Warring States period (475 BC-221 BC) city named Da Liang. The next major occupation was during the Han and Tang dynasties, but it was not until the capital was relocated to Kaifeng during the Song dynasty that the city reached its zenith. During the Song dynasty, nearly a million people lived in Kaifeng, making it one of the world's most populous cities at that time. However, the Song dynasty abandoned the city in AD 1128 after the governor of Kaifeng failed to stop the advancing Jurchen army by breaching the dikes along the Yellow River[7]. The next substantial occupation phase dates to the Ming dynasty when Kaifeng was reduced to a minor capital. In AD 1642, the dikes along the Yellow River were intentionally breached, releasing the Yellow River's flood waters that destroyed Kaifeng and killed around 300,000 people[8]. After this disaster, the city was abandoned until AD 1662 when it was rebuilt under the orders of the Qing dynasty Kangxi Emperor. Another major flood destroyed parts outside the main wall of Kaifeng in AD 1841, when an exceptional storm caused the Yellow River to breach its levees.

During the Ming dynasty, when droughts destroyed the livelihoods of millions of peasants, Li Zicheng and his peasant army sieged Kaifeng for months. During the siege, Li Zicheng and his army or the Ming dynasty troops breached the dikes along the Yellow River, causing the AD 1642 flood that destroyed Kaifeng. With almost no time to respond, the floodwaters engulfed the entirety of Kaifeng, causing massive amounts of devastation.

2. Translated historical records

2.1 *The AD 1642 Yellow River flood*

The historical record of the AD 1642 flood is primarily derived from the *Ming Shilu*, literally meaning *Veritable Documents of the Ming*. This document was composed from the beginning of the Ming dynasty to the end of the dynasty. Before the emperor died, he selected a historian to write the dynastic history. This is one of the main documents used to reconstruct Ming dynasty history. In the *Book of Chongzhen*, the last emperor of the Ming dynasty, there is a passage that specifically details the flood at Kaifeng:

"On September 23, Lu Ruoteng, the Superintendent of Coastal Defense of Zhejiang reported: "I arrived in Linqing in August and saw that Tian Xingguo, an internal official in court linked 24 boats together, and commanded more than 900 people for service. His fleet disturbed the courier stations and jammed the water gates." The Emperor ordered the Silijian eunuch to take over Tian's postal *fu* (an object that gives him the privilege of using courier stations). The levee of the Yellow River breached near Kaifeng and afterwards the city was taken over (by the rebellions led by the peasant Li Zicheng). Five days earlier, the levee first breached near Zhujiazhai (4 km north of Kaifeng) and flooded the northern portion of Kaifeng. Shortly afterward, the entire city was flooded. The King of Zhou, Gongxiao and other kings escaped to Cizhou because Circuit Censor Wang Han welcomed them with boats. Governor Gao Mingxing and other officials all fled to the north of the Yellow River. Lower officials and soldiers fled to different places. Several hundred thousand people drowned. The city walls were severely damaged, and the government and private buildings inside were all inundated. The rebellion did not suffer much loss because they were encamped on high terrain. The cause of the flood was that Huang Shu, the Judiciary Official of Kaifeng, dug a canal to channel water as the water stage of the Yellow River rose in the fall, which accidently led to the outflow of water. Most of the flood flowed to the Si River and the Huai River and became separated from the main channel of the Yellow River. Both Pizhou and Bozhou were flooded. The entire family of Liang Kecong, former vice head of the Court of Imperial Sacrifices drowned. Huang De, an Assistant Commander, beat the roving rebels at Xiaoshi in Qianshan."

> *Chongzhen Shilu,* the fifteenth year of the Chongzhen era, September 23 (October 16, Gregorian calendar, AD 1642)

2.2 *The AD 1841 Yellow River flood*

According to the *Shuizai Bianliang Jilue (Flood Disaster at Kaifeng)* written by an intellectual whose penname was *Tongding Sitong Jushi*, the Yellow River started to flood in August 1841 and did not stop until March 1842[9]. A recent study uses the geographic information found within this document to map the flood event only entered the city for a short period of time before it then surrounded the city walls, in agreement with the stratigraphic records found at Xinzhengmen and Yongning Wangfu[10].

"Outside the city, the Yellow River is boundless and the villages are not seen. Along the moat, the willow trees were all around, and all the branches were gone."

> *Shuizai Bianliang Jilue (Flood Disaster at Kaifeng)*

3. Excavated sites at Kaifeng

Until recently, almost all knowledge about ancient Kaifeng has come from dynastic chronicles and inscriptions found on stelae. The results of archaeological excavations, now published in several Chinese language journals and monographs, provide a detailed reconstruction of certain areas of the city that have remained intact underneath the modern surface of Kaifeng[11,12]. Although the history of urban archaeology in China extends back into the 1930s', excavations at Kaifeng did not begin until the 1980s' when archaeologists found the first evidence of the ancient cities buried underneath the modern city[13-15]. For the most part, excavations are conducted under salvage conditions by the Kaifeng Institute of Archaeology and Cultural Relics as a result of Kaifeng's breakneck pace of economic development and modernization. These excavations are primarily focused on several locations of critical importance to the Song and Ming dynasty history of Kaifeng, such as the palaces, bridges, and walls of the city.

Excavations in the 1980s' revealed that the plan of the Old Town of Kaifeng has remained relatively unaltered since the Tang dynasty[11]. Modern Kaifeng's "Imperial Street" (Zhongshan Road) was first built during the Tang dynasty and has remained in its current position through five different dynasties, the Song, Yuan, Ming and Qing. Archaeological excavations at the northern section of Zhongshan road also revealed one of the main thoroughfares, Zhouqiao, which is located 3 m underneath the modern Zhongshan road. Zhouqiao was an important bridge that was located on the central axis of Zhangzhou City into the city after the Luan River Circle. Archaeological excavations suggest that the bridge was rebuilt during the Ming dynasty in the exact same location as the earlier bridge.

Throughout the modern city boundaries of Kaifeng, urban archaeologists have found widespread evidence of the AD 1642 flood at several sites, including the sites discussed in this paper[16]. Here, we provide additional background details, stratigraphic descriptions, and information on the radiocarbon sampling for each site mentioned in the paper at Xinzhengmen, Yongning Wangfu, Dichen Xiyuan, Xinjiekou, and Yulongwan. We also present a summary table with all the collected chronological information from these excavations (Supplementary Table 2).

3.1 *Xinzhengmen (S1)*

Coordinates: 114° 18' 15.70" E, 34° 47' 42.38" N

Xinzhengmen is the western main gate of the Song dynasty city that was only for the use of the emperor and his ministers. The Song dynasty gate is 160 m wide and 100 m long and covered a total of 16,000 m[17,18]. This style was first adopted during the Northern Song dynasty and continued in the same tradition for the next thousand years. The 2,100 m^2 excavation at Xinzhengmen has recently concluded and revealed many architectural features on the Song, Yuan, and Qing dynasty land surfaces. Here, the archaeological features of the Song dynasty gate and roads are intact and superimposed by later dynastic gates and roads in the same orientation and position. Approximately 8 m deep, the large scale of the Xinzhengmen excavations has allowed archaeologists to uncover evidence of the main gate, roads, agricultural fields, wells, houses, middens, hearths, and the stratigraphic record of Kaifeng's urban history from the Song dynasty to the Qing dynasty.

Excavations in the north and south revealed the Qing dynasty occupation horizons. The northern section of the excavation is about 4 m deep and exposes a suburb of Qing dynasty Kaifeng, containing one well, ridged fields, and two houses with courtyards. The courtyard contained many artifacts left *in situ* by the house's inhabitants. These households were buried by a Yellow River flood in AD 1841.

In the eastern part of the excavation, the unit is approximately 8 m deep and contains the remains of the Song dynasty city wall and gate. The wall is made from *hangtu* (rammed earth). There are also living surfaces and building foundations on both sides of the city wall. The foundations of the Song dynasty city wall are 23 m wide and are comprised of yellow-brown clay *hangtu*.

Both sections contain evidence for Yellow River flood events from AD 1642 and AD 1841. In the southern section, a clay filled incised channel dates to the Ming dynasty flood of AD 1642. In the northern section, the Qing dynasty households were abandoned and buried by a large Yellow River flood event in AD 1841. The AD 1841 flood deposits shown in profile reveal many laminated beds that likely represent episodic flooding that occurred over many months, roughly in accord with the description found in historical documents[19].

See the published excavation report for more details[18].

Stratigraphy

The section is about 7.5-8 m deep (65-72 m above sea level) and can be divided into nine layers from top to bottom.

S1-1 (0-60 cm): Disturbed modern layer, 60 cm thick.

S1-2 (60-200 cm): Qing dynasty, 140 cm thick, light yellow, fine sand 2.5Y 6/3.

S1-3 (200-320 cm): Qing dynasty, 120 cm thick, light yellow, fine sand 2.5Y 6/3, the particles are coarser than the preceding two layers and are deposited in horizontal beds.

S1-4 (320-350 cm): Qing dynasty, 30 cm thick, gray-yellow fine to medium sand, 2.5Y 7/6, with horizontal bedding.

S1-5 (350-400 cm): Qing dynasty, 50 cm, yellow silt, 2.5Y 6/6, looser than previous layers.

S1-6 (400-500 cm): Qing dynasty, 100 cm thick, contains cultural material like stones and bricks, gray-yellow silt 10YR 6/1.

S1-7 (500-590 cm): Ming dynasty, central part of the gully alluvial silt, 90 cm thick, reddish brown clay (mud), 10YR 4/3, good cementation, there is an ancient road on both sides of the ditch.

S1-8 (590-790 cm): Yuan dynasty, 200 cm thick, with an ancient road.

S1-9 (790 cm -): Jin-Song dynasty, bottom of the excavation.

Radiocarbon sampling

We collected two radiocarbon dates from the eastern profile at Xinzhengmen, one at ca. 66.1 m above msl (Beta 535781) and another at 68.5 m above msl (Beta 535783). The sample from

context L14, S1-8 (Beta 53781) is located directly beneath the incised flood channel and returned a date of 950 ± 30 BP, calibrated to AD 1024-1155 (95.4%). The sample from context H231, S1-6 (Beta 535781), a meter and a half above the flood deposit, returned a date of 220 ± 30 BP, calibrated to AD 1735-1806 (46.2%), AD 1642-1854 (37.9%), and AD 1933- post 1950 (11.3%). These two sampling locations are below and above the river channel sediments expected to be associated with the AD 1642 flood. The date range provides a bracketed brackets the flood deposit and constrain it to around AD 1642. We also collected a sediment sample from the AD 1642 channel, S1-7, for an organic matter date which returned an anomalously old radiocarbon age of 7120 ± 30 BP (Beta 535782).

Another sample for radiocarbon dating was collected from a buried tree located on the northern profile within the flood deposit that is presumed to date to AD 1841 (Beta 500051). The sample returned a date of 130 ± 30 BP, which calibrates to AD 1798-1894 (42.4%), AD 1674-1778 (95.4%), AD 1905-1942 (14.9%). This tree was buried by the AD 1841 flood and returned a date roughly corresponding with our expectations.

3.2 *Yongning Wangfu (S2)*

Coordinates: 114° 20’ 11.08” E, 34° 47’ 50.69” N

Recent excavations in Kaifeng have revealed several different parts of the Ming dynasty *Wangfu*[20]. Built in the center of old Kaifeng near the old Song dynasty palace, the Kaifeng *Wangfu* were central pillars of the Ming dynasty’s authority in peripheral cities and a symbol of elite culture. Kaifeng had several *Wangfu* but their role in governing and establishing cultural institutions are poorly documented in the historical record[21]. In recent years, archaeologists working in Kaifeng have excavated a few of these Ming dynasty *Wangfu*, providing unprecedented insights into elite culture at the eve of the Yellow River flood that destroyed almost all of Kaifeng in AD 1642.

In 2017, the Kaifeng Institute of Archaeology and Cultural Relics discovered a *Wangfu* palace named Yongning Wangfu. Here, archaeologists excavated over 4,000 m^2 and revealed the layout of the palace, recovering over a 1,000 sets of Ming dynasty porcelain, kitchenware, copperwares, and many other architectural artifacts. The palace itself is a series of three interconnected courtyards arranged along the central axis of Kaifeng. The entire group of buildings is oriented north-south and is about 200 m long and 115 m wide. The central axis contains the main buildings of the *Wangfu*, including the walled gate to the compound, an internal gate to the front hall (Yin’an Temple), and the back area with sleeping quarters. To the north of the back hall is a garden with the depressions of former ponds. Each main building is connected by a road or a ramp along the central axis. The AD 1642 Yellow River flood deposit that buried this princely house is approximately 3 m deep, depending on the location, and primarily composed of reddish silt to silty clay, typical of Yellow River flood deposits.

No formal report has yet been published on this site, but descriptions of the excavations have been published[20].

Stratigraphy

S2-1 (0-90 cm): Qing dynasty cultural layer, 90 cm thick, silty clay, 10YR 7/2.

S2-2 (90-170 cm): Scouring during the AD 1642 flood, 80 cm thick, silty clay horizontal laminated beds, predominately mainly silt, about 10 cm silt and 2 cm clay superimposed on each other, the silt is yellow 2.5Y 7/4 and the clay is reddish brown 10YR 6/3.

S2-3 (170-220 cm): Scouring during the AD 1642 flood, 50 cm thick, silty clay horizontal laminated bedding with undulations, the upper 10 cm is predominately silt that becomes more clayey with depth. Layers 2 and 3 layers are broken by the Qing dynasty re-occupation of Kaifeng. Archaeologists found a stela inscribed with the date of the 34th year of the Kangxi emperors' reign (*Chao Jin ding tai he shan beiji*), or AD 1695, indicating that people reoccupied the city around 50 years after the AD 1642 flood (68.8 m amsl).

S2-4 (220-390 cm): Scouring during the AD 1642 flood, 170 cm, silty clay with bedding indicating highly turbid flow, gray sand layer containing clay with a spiral structure is abundant throughout this layer. The lower part of the bed is clay. Silty sand 10YR 5/6, clay 10YR 4/1.

Radiocarbon sampling

In total three radiocarbon samples were taken. The skeletonized individuals selected for sampling came from excavation Trench 15, Building 9 (#86 and #92) (Beta 5335791 and 5335791). We collected one incisor from each individual for radiocarbon dating. Individual #86 returned a radiocarbon date of 390 ± 30 BP, which calibrated to AD 1440-1524 (69.2%), AD 1571-1630 (25.7%), AD 1559-1562 (0.5%), roughly corresponding with the AD 1642 flood event. Individual #92 returned a radiocarbon age of 330 ± 30 BP, which calibrated to AD 1477-1642 (95.4%). The final report on the bioarchaeology of these individuals is still in progress.

Another sample was taken from a wooden column. This wood sample returned a date of 340 ± 30 BP (Beta 500052), calibrated to AD 1470-1640 (95.4%).

3.3 *Dichen Xiyuan (S3)*

Coordinates: 114° 20' 30.51" E, 34°48' 13.71" N,

The site is located in the north of Kaiyuan Community, No.1 Longting Lake, north of Xiaoqiong Street, east of Longtinghu West Road, and southwest from the first affiliated hospital of Henan University. The site covers an area of approximately 3,700 m^2 and is part of a salvage excavation from a construction project.

In 2016, the Kaifeng Institute of Archaeology and Cultural Relics found a large area with ancient architectural remains about 5.5-8 m deep. In mid-June 2019, the Kaifeng Institute of Archaeology and Cultural Relics started rescue excavations at the proposed construction area.

At the time of writing, excavations are still on-going. The area to be excavated is about 1,600 m^2. The main findings includes six building foundations that date to the late Ming dynasty and four kilns from the early Ming dynasty. The silt layer that buries the site is approximately 5 m thick and represents the AD 1642 flood. Of particular note, archaeologists have found a series of kilns at the site that are likely related to an early Ming dynasty glass foundry.

No formal report has yet been published on this site.

Stratigraphy

No precise measurements have yet been taken at the site. We reconstruct the stratigraphy based on photographic evidence.

S3-1 (0-250 cm): Topsoil and modern and recent historic (Qing dynasty) deposit. Light gray, silt.

S3-2 (250-500 cm): Ming dynasty deposit, many artifacts and buildings buried approximately 300 cm deep. Light brownish red silt and silty clay.

Radiocarbon

We collected a piece of bark from a buried tree in a courtyard of a building (Beta 535785). The sample returned dates of a date of 280 ± 30 BP, calibrated to AD 1512-1600 (54.3%), AD 1616-1666 (38.3%), AD 1784-1795 (2.1%), and AD 1498-1502 (0.6%).

3.4 *Xinjiekou (S4)*

Coordinates: 114° 20' 40.31" E, 34° 48' 0.19" N

This Ming dynasty *Wangfu* for the Prince of Zhou is located at Xinjiekou, north of Longting Park by about 500 m. In March 2014, the Kaifeng Institute of Archaeology and Cultural Relics carried out a salvage archaeological excavation here, in total around 400 m^2. The archaeologists found material culture mostly dating to from the late Ming dynasty to the late Qing dynasty. The Ming dynasty cultural remains include one courtyard and several houses with collapsed walls, as well as lacquerware, porcelain, pottery and other daily necessities found inside the buildings. The excavation results showed that this site was the location of the *Dianyi Suo* (Office of the Rites) and was destroyed in the AD 1642 flood[22]. Several wood plaques were found at the bottom of these excavations with reign dates that serve as temporal anchors for the site.

See the report for full details[22].

Stratigraphy

The section is around 2.4-4 m deep. The lowest layer excavated at Xinjiekou is the Ming dynasty construction. Excavations could not proceed any farther due to groundwater.

S4-1 (0-25 cm): Disturbed layer. The soil is hard and 20-25 cm thick, contains many porcelain and brick fragments that date to the Republic of China.

S4-2a (25-100 cm): Hard soil, 75-160 cm thick. This layer contained many porcelain, pottery, and brick fragments along with slag and copper coins that date to layer to the middle / late Qing dynasty.

S4-2b (25-100 cm): Yellow-brown soil, slightly loose, 75 cm thick. This layer contains porcelain tiles, pottery pieces and a small number of animal bones that likely date to the end of the Qing dynasty.

S4-3 (100-120 / 232 cm): Reddish-brown clay, hard and sticky, with yellow sand inclusions, 120-232 cm thick. Few objects in this layer, only a small number of wooden blocks and tiles. This is the AD 1642 flood deposit.

S4-4 (120-240 / 370 cm): Gray-black, loose soil 80-120 cm thick, contains bricks and tiles, wooden beams, porcelain, and copper coins. Judging from the relationship between the stratum and the characteristics of the unearthed objects, this layer is the accumulation of culture material dating to the late Ming dynasty. A courtyard with five collapsed roofs and the remains of the house foundations were also unearthed. Archaeologists also unearthed wooden plaques dating to the tenth year of Emperor Chongzhen's reign (AD 1637), providing a reliable chronological anchor for this deposit (artifact number JZ9: 308).

3.5 *Yulongwan (S5)*

Coordinates: 114° 21' 19.09" E, 34° 47' 7.57" N

Yulongwan is about 440 m east of the east wall of Kaifeng during the Ming and Qing dynasties[23]. In April 2016, the Kaifeng Institute of Archaeology and Cultural Relics carried out rescue excavations and opened up a total area of 970 m^2. They found three building bases that date to the late Ming dynasty. Due to the high groundwater level in the area, after exposing the cultural layer of the late Ming dynasty, it was impossible to further excavate the site.

Building 1 is located in the southeast of Trench 1 and three walls were discovered. Building 2 is located in the southwest of Trench 1. Building 3 is located in the western half of the excavation area and has a large courtyard that can be divided into a front and back yard. The front and back yards are separated by a corridor. The main artifacts in Building 3 include a courtyard gate, four roads and one corridor.

Many coins were found at the site, and the latest dates to the *Chongzhen Tongbao* era, which was in the late Ming dynasty. Many blue and white porcelains found at the site have late Ming characteristics. By calculating a date recorded on an inscribed brick (artifact number F3-8:664), the latest date for the site is around January 11 to February 8, AD 1641, that is, the thirteenth year of Emperor Chongzhen. According to these points of evidence, along with the radiocarbon dates collected from two individuals, the age of the site dates to the end of the Ming dynasty and the AD 1642 flood.

See the report for full details[23].

Stratigraphy

All stratigraphic descriptions come from the full report's detailing of a core taken at the site. All depths are approximate.

S5-1 (0-50 cm): Topsoil, about 20-50 cm thick. The soil is hard, gray-brown, and contains modern garbage and brick fragments.

S5-2 (50-300 cm): The cultural layer of the Qing dynasty, about 250-280 cm thick. The soil is loose, gray-brown, and contains many bricks, pottery pieces, and a small amount of blue-and-white porcelain pieces.

S5-3 (300-600 cm): A silt layer about 300-350 cm thick. The soil is soft and pure, reddish brown with no cultural relics. The Ming dynasty remains around found at about 300-400 cm deep. The layer has laminated bedding indicating flooding.

S5-4 (600-10000 cm): The Tang to Ming dynasty archaeological horizon. It is 350-400 cm thick. The soil is loose, gray, and it contains cyan and white glazed tiles, pottery pieces, bones and many other artifacts.

S5-5 (1000-1100 cm): A layer of silty clay about 10-11 m deep, 100 cm thick. The soil is soft and yellow with no artifacts.

Layer 6 (1100 cm -): Slightly hard, yellow soil with sandstone inclusions.

Radiocarbon sampling

We sampled two individuals found from this flood from Trench 1, *Jian* 6, eastern wall (Beta 535789) and Trench 2, Building 1, *Jian* 11 (Beta 535790). Individual *Jian 6* returned an age of 340 ± 30 BP, which calibrates to AD 1470-1640 (95.4%). Individual *Jian 11* returned a radiocarbon age of 330 ± 330 BP, which calibrates to AD 1477-1642 (95.4%). The final bioarchaeological report on these individuals is still in progress.

4. Supplemental References

1 Wheatley, P. *The pivot of the four quarters: a preliminary enquiry into the origins and character of the ancient Chinese city*. (Edinburgh University Press, 1971).

2 Dodgen, R. A. Hydraulic Evolution and Dynastic Decline: The Yellow River Conservancy, 1796-1855. *Late Imperial China* **12**, 36-63, doi:10.1353/late.1991.0009 (1991).

3 Milliman, J. D., Yun-Shan, Q., Mei-E, R. & Saito, Y. Man's Influence on the Erosion and Transport of Sediment by Asian Rivers: The Yellow River (Huanghe) Example. *The Journal of Geology* **95**, 751-762, doi:10.1086/629175 (1987).

4 Chen, Y., Overeem, I., Ketter, A. J., Gao, S. & Syvitski, J. P. Modeling flood dynamics along the superelevated channel belt of the Yellow River over the last 3000 years. *Journal of Geophysical Research: Earth Surface* **120**, 1321-1351 (2015).

5 Chen, Y., Syvitski, J. P., Gao, S., Overeem, I. & Kettner, A. J. Socio-economic impacts on flooding: a 4000-year history of the Yellow River, China. *Ambio* **41**, 682-698, doi:10.1007/s13280-012-0290-5 (2012).

6 Liu, C. Kaifeng diqu chuantong diming zhong yunhan de kaoguxue xinxi kaoshi [An interpretation of archaeological information contained in traditional place names in the Kaifeng Area]. *Kaifeng Daxue Xuebao* **31**, 26-32 (2017).

7 Lamouroux, C. in *Sediments of time: Environment and society in Chinese history* (eds Mark Elvin & Cuirong Liu) 545-584 (Cambridge University Press, 1998).

8 Wu, P., Liu, D., Ma, J., Gu, L. & Tong, J. Reconstructing the man-made Yellow River flood of Kaifeng City in 1642 AD using documentary sources. *International Journal of Disaster Risk Reduction* **41**, 101289 (2019).
9 Li, J. W., Wang, S. Z. & Li, T. B. *Bianliang Shuizai Jilue [A description of flood disasters at Kaifeng]*. (Henan Daxue Chubanshe, 2006).
10 Wu, P. F., Lu, J. & Ma, J. H. 1841 nian huanghe jueyi weikun Kaifengcheng de kongjian zaixian yi yuanjin fenxi [Spatial reconstruction of the Yellow River flood seiged Kaifeng in 1841 and the reasons for the flood]. *Journal of Henan University (Natural Sciences)* **44**, 299-305 (2014).
11 Qiu, G., Li, H. & Liu, C. *Kaifeng kaogu faxian yu yanjiu*. (Zhongzhou Guji Chubanshe, 1998).
12 Liu, C. *Kaogu Kaifeng [The archaeology of Kaifeng]*. (Henan Daxue Chubanshe, 2006).
13 Qiu, G. Kaifeng gucheng de xingfei yu kantan [Revitalization and exploration of Kaifeng]. *Lixue Yuekan* **1992**, 97-101 (1992).
14 Qiu, G. & Sun, X. Beisong Dongjing waicheng de chuban kantan yu shijue. *Wenwu* **1992**, 52-61 (1992).
15 Whitehand, J. & Gu, K. Research on Chinese urban form: retrospect and prospect. *Progress in Human Geography* **30**, 337-355 (2006).
16 Ge, Q. Kaifeng cheng Mingqing shuizai de kaoguxue guancha [Archaeological Observation of Floods in Kaifeng City]. *Huaxia Kaogu* 11 (2019).
17 Ge, Q. S. Beisong ducheng xinzhengmen yizhi yu kaifeng "cheng luo cheng" [The Xinzheng Gate Site in the Northern Song Dynasty capital city Kiafeng "cheng luocheng"]. *Dazhong Kaogu* **10**, 36-40 (2014).
18 Ge, Q., Liu, H. & Wang, S. Henan Kaifeng Beisong Dongjingcheng Shuntianmen yizhi 2012-2017 nian kantan fajue jianbao [Excavation report at Henan Kaifeng Beisong Dongjingchen Shuntianmen site from 212-2017]. *Huaxia Kaogu*, 13-41 (2019).
19 Ye, F., Fang, X. & Li, F. Response and recovery measures for two floods in north China during the nineteenth century: a comparative study. *SpringPlus* **5**, 1985 (2016).
20 Wang, S. Henan Kaifeng fajue Mingdai Yongning Wangfu yizhi [Excavations at Henan Kaifeng Ming dynasty Yongning Wangfu site]. *Zhongguo Wenwubao* **8**, 1 (2019).
21 Robinson, D. M. Princely courts of the Ming dynasty. *Ming Studies*, 1-12 (2012).
22 Wang, S. Henan kaifeng xinjiekou ming zhou wangfu guanshu yizhi fajue jianbao [Excavation bulletin of the Ming dynasty Wangfu Guanshang site, Xinjiekou, Kaifeng, Henan]. *Wenwu* **2017**, 39-72 (2017).
23 Wang, S. *et al.* Kaifeng Yulongwan xiaoqu Mingdai jianzhu yizhi de fajue [Excavation of Ming Dynasty Architectural Sites in Yulongwan Community, Kaifeng]. *Huaxia Kaogu*, 8 (2019).

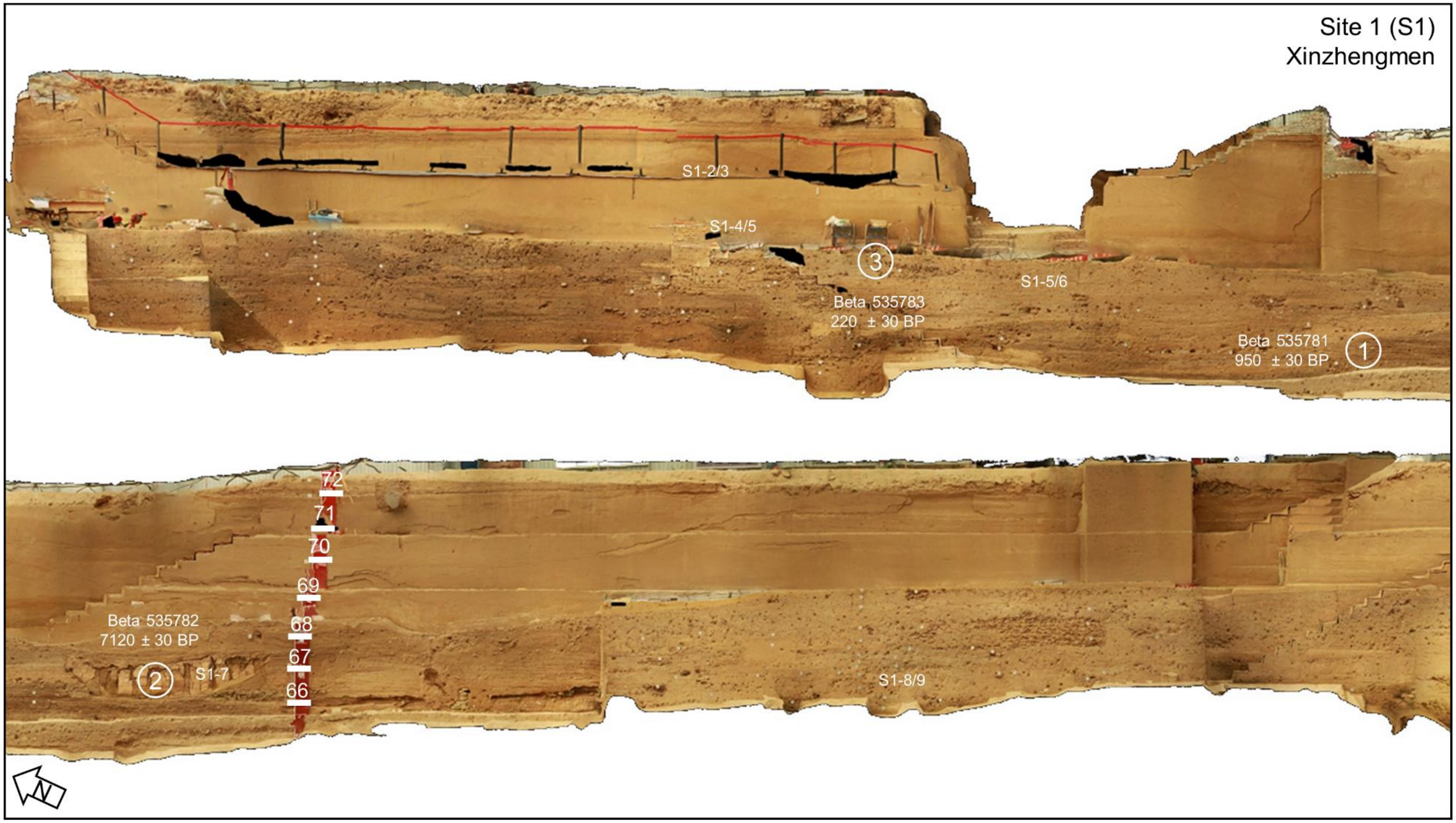

Supplementary Figure 1. Xinzhengmen western profile. Locations of radiocarbon samples numbered from bottom to top, 1 (Beta 535783), 2 (Beta 535782), 3 (Beta 535781) and meters above mean sea level marked on the profile.

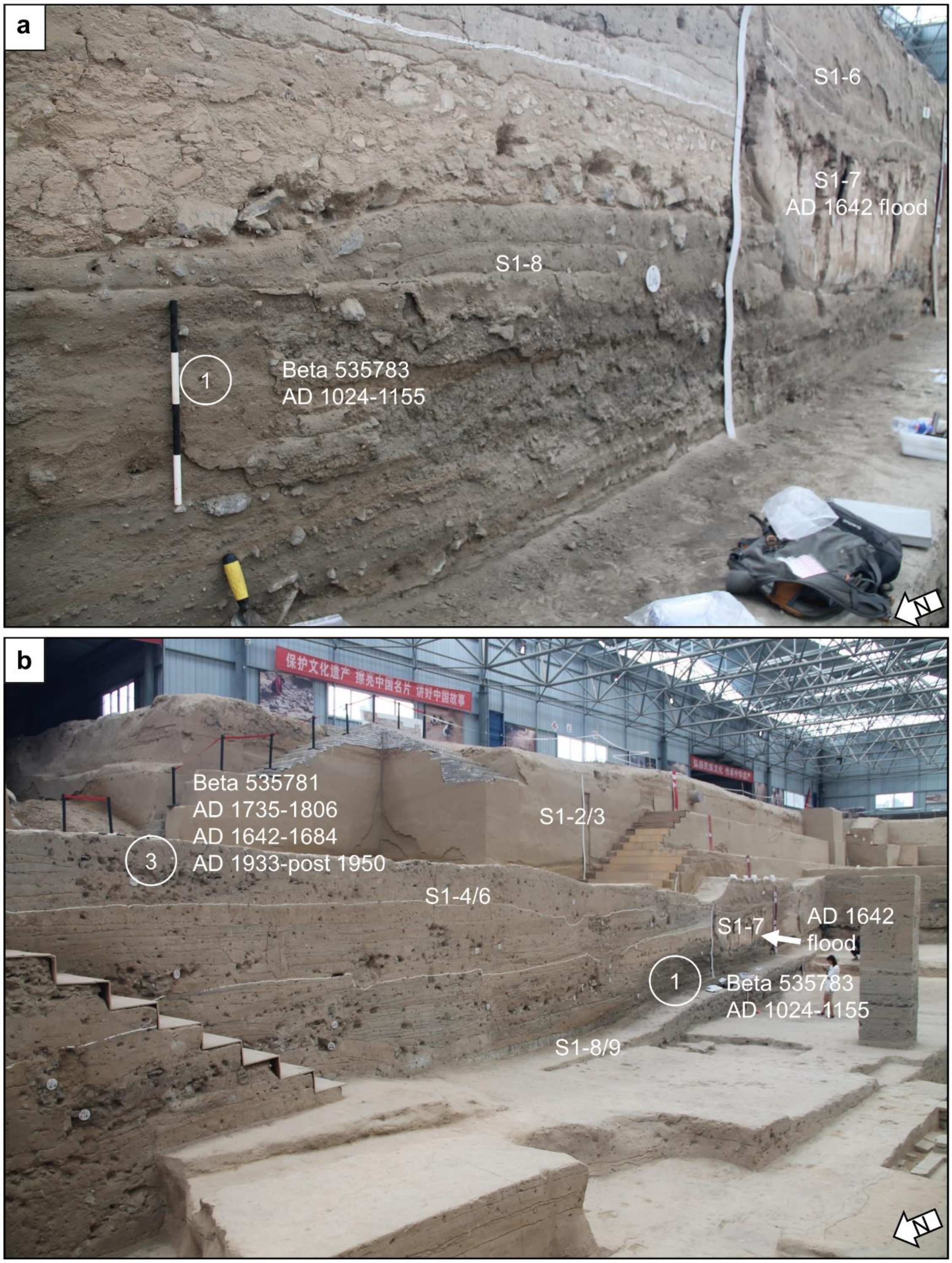

Supplementary Figure 2. Xinzhengmen western profile. a, photograph of sample Beta 535783 within context of AD 1642 flood, and the deposit incising through the above road. b, photograph of both Beta 535783 and Beta 535781, showing their relative stratigraphic position in relation to the AD 1642 Yellow River flood.

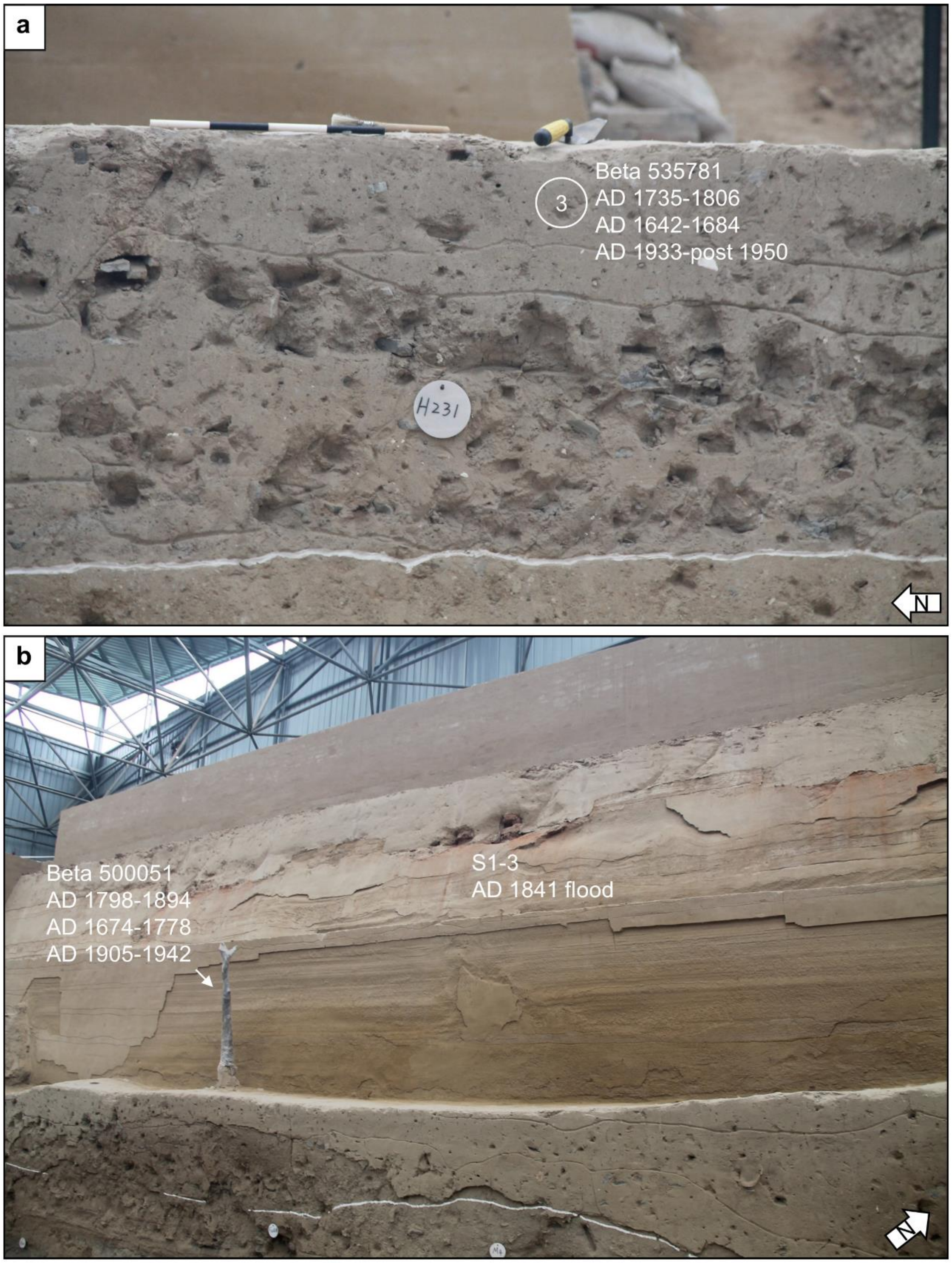

Supplementary Figure 3. Xinzhengmen radiocarbon sample locations. a, close up photo of the sampling location of Beta 535781. b, radiocarbon sample of the sampled buried tree Beta 500051, note the laminations within the AD 1841 Yellow River deposit.

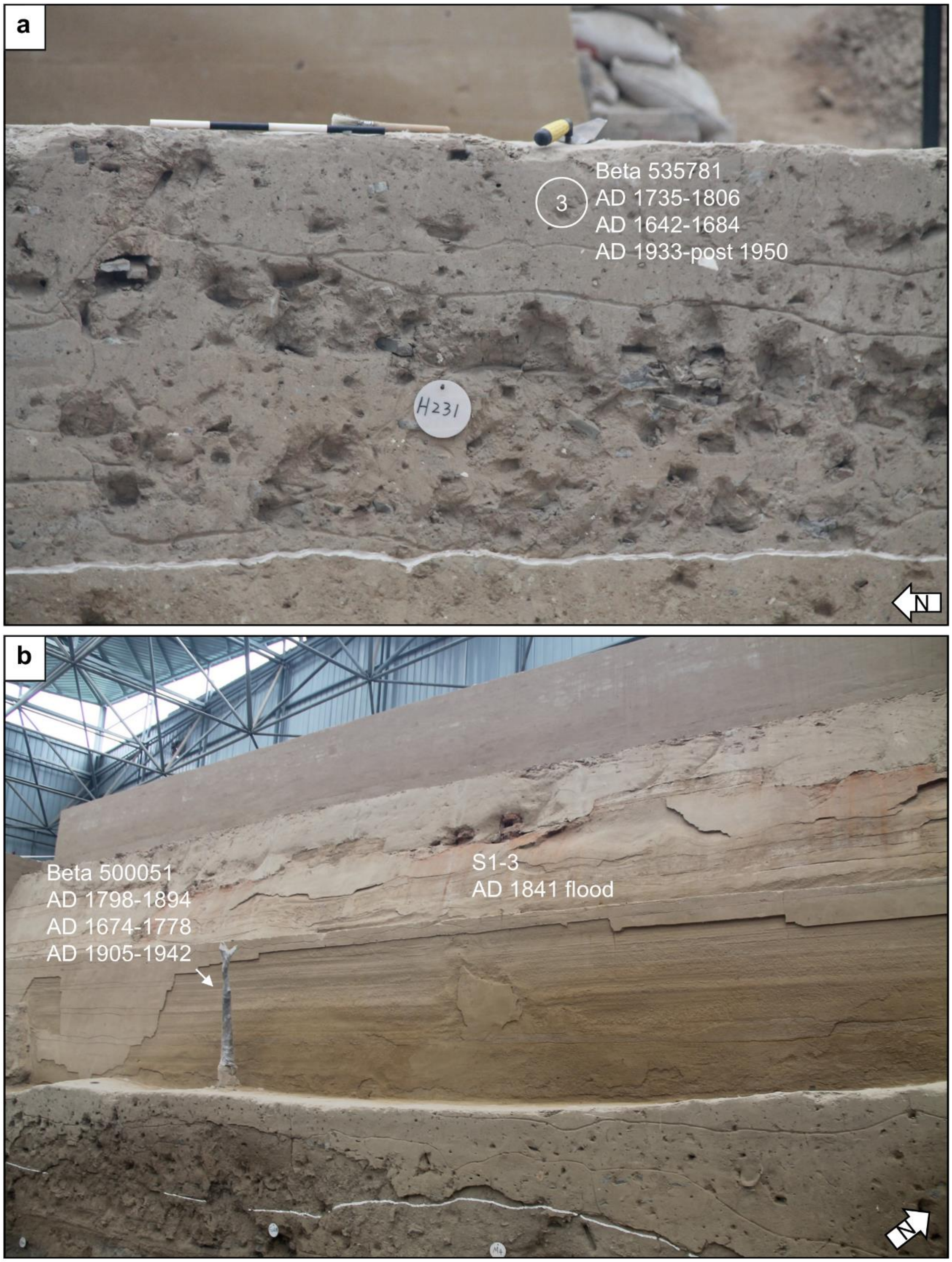

Supplementary Figure 4. AD 1642 Yellow River flood deposit, eastern profile. a, photo of the AD 1642 flood deposit showing that the upper section is predominately a reddish brown silty clay loam. b, underneath the reddish brown silty clay loam deposit are the sandy lag deposits of the flood channel with coarser gravel inclusions.

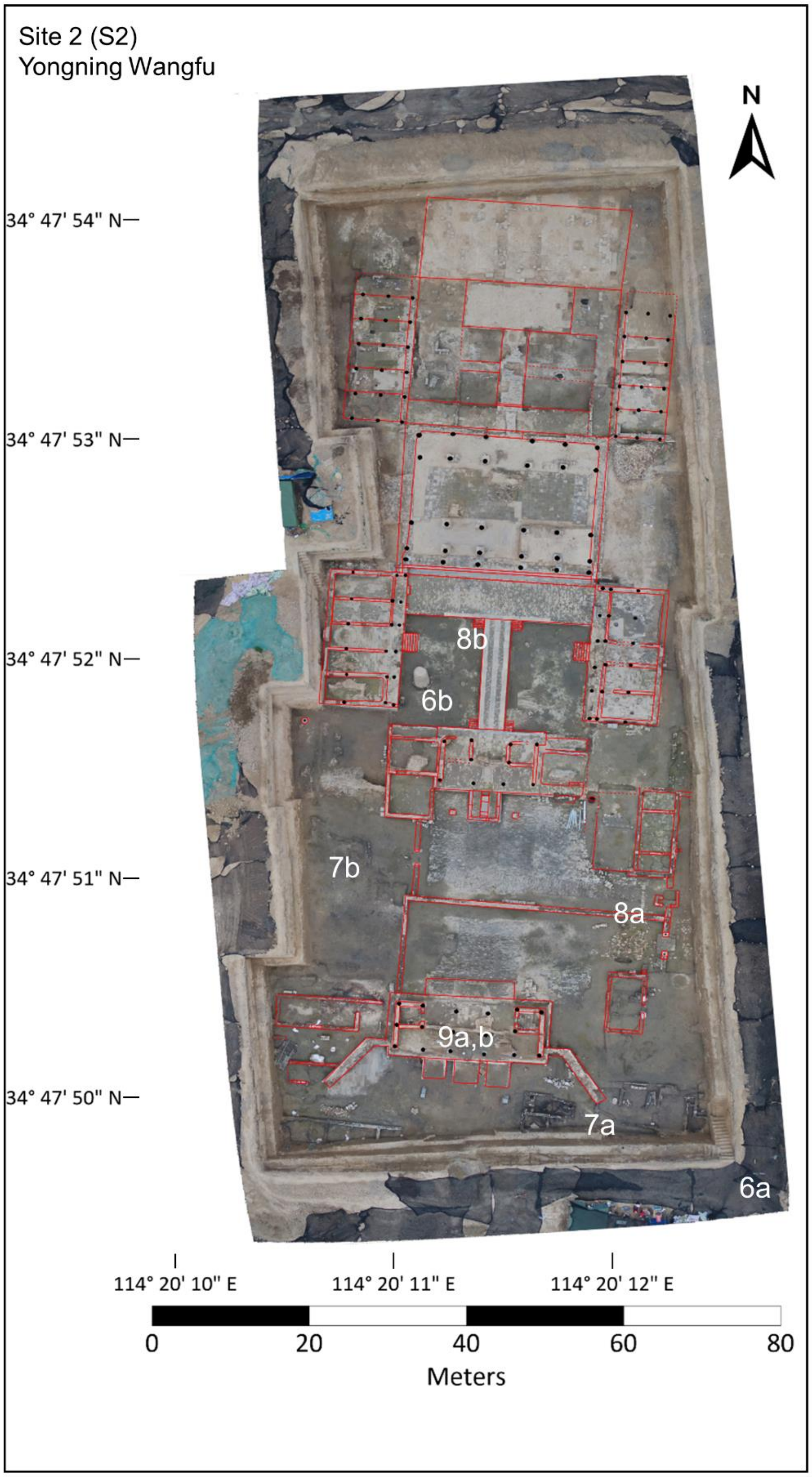

Supplementary Figure 5. Plan view of Yongning Wangfu with locations of following figures marked on the map.

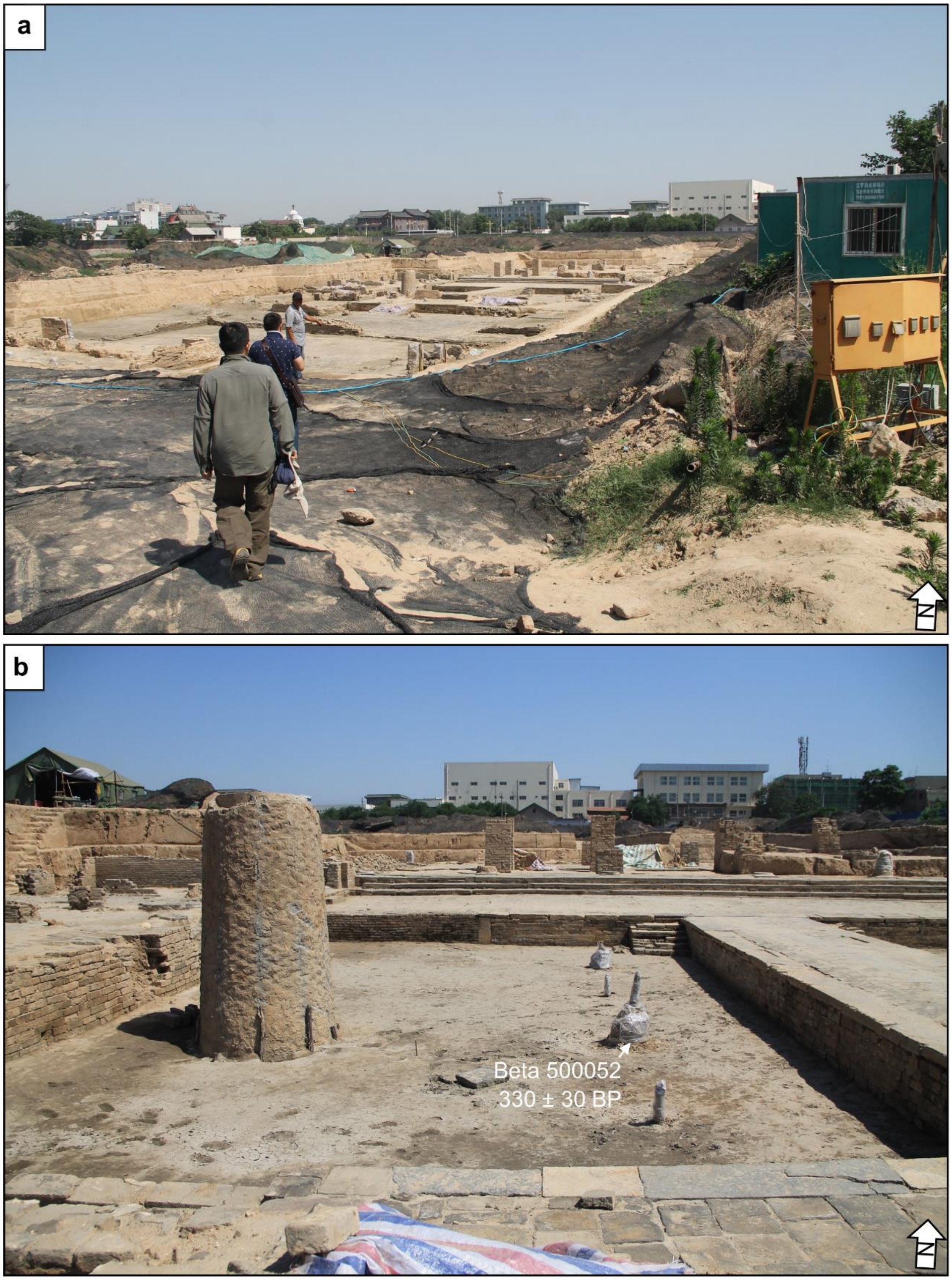

Supplementary Figure 6. a, Photo of excavations at Yonging Wangfu. b, main courtyard of Yongning Wangfu with locations of buried trees.

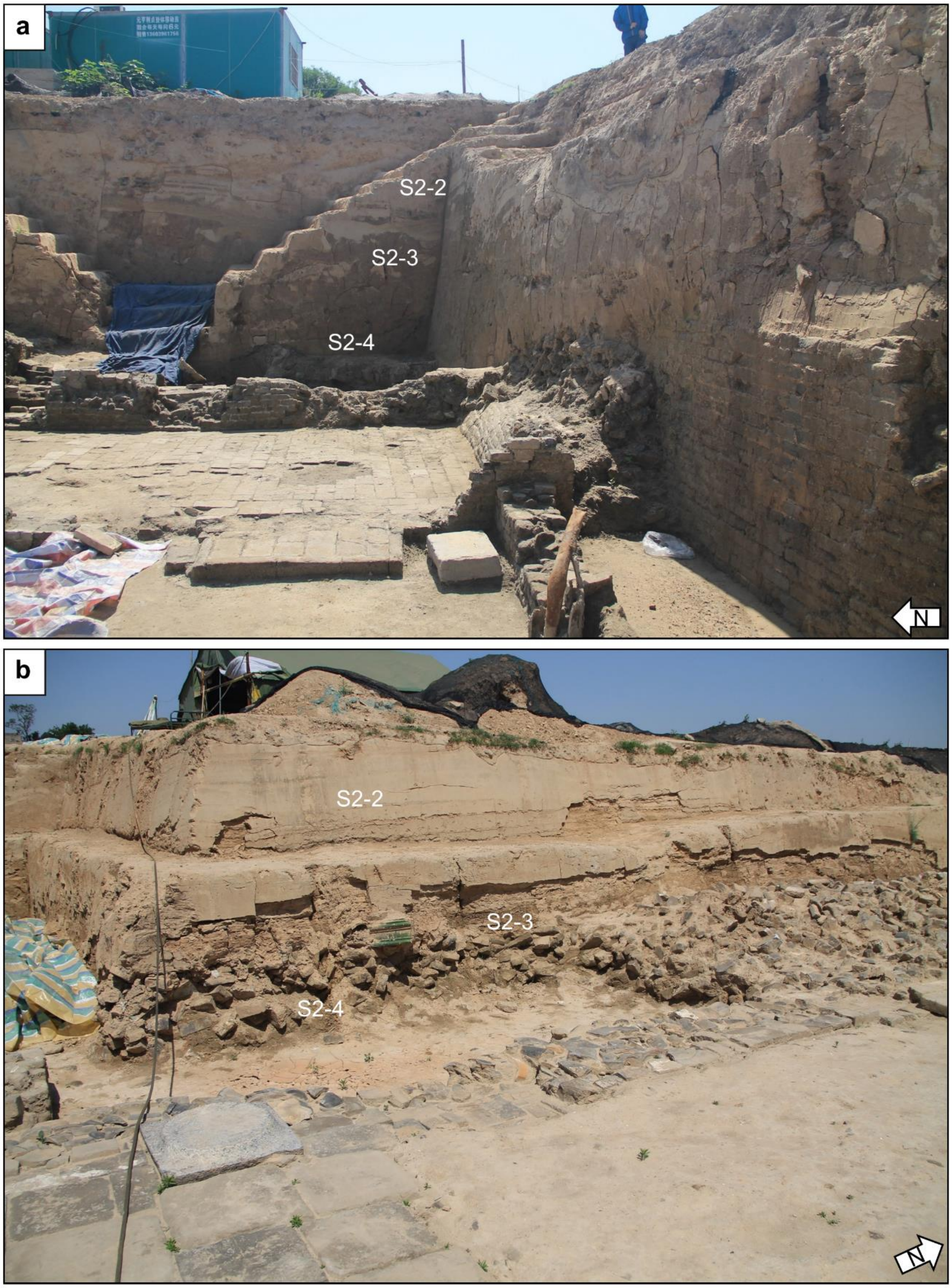

Supplementary Figure 7. a, Southeastern profile of Yongning Wangfu. b, destroyed brick walls at Yongning Wangfu.

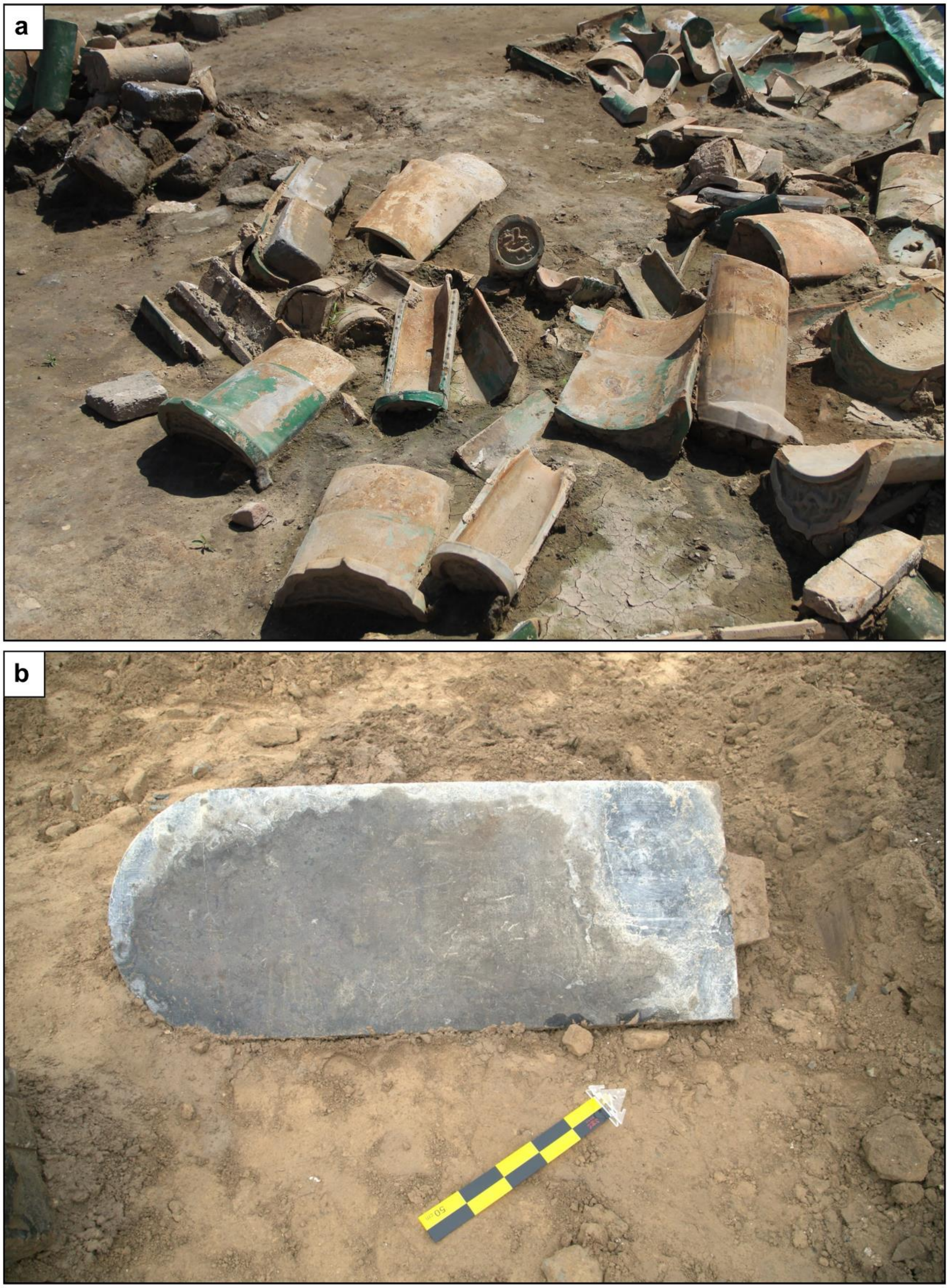

Supplementary Figure 8. a, Ming dynasty Chinese dragon on the *wadang* (roof tile). b, stelae with inscription from the 13th year of the Kangxi emperor, or AD 1695.

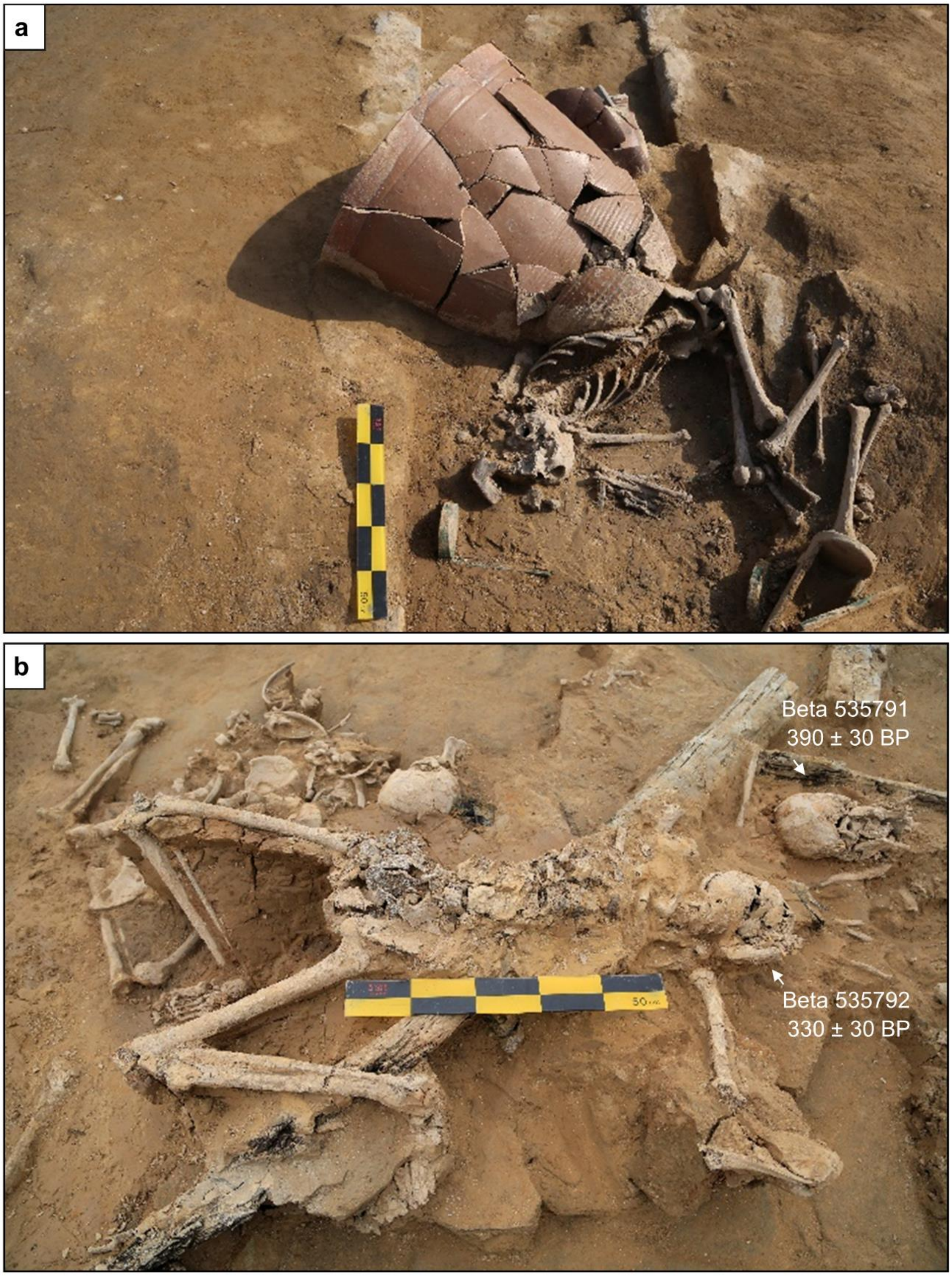

Supplementary Figure 9. a, Individual with ceramic vessel intruding into abdomen. b, at least three individuals with flood debris.

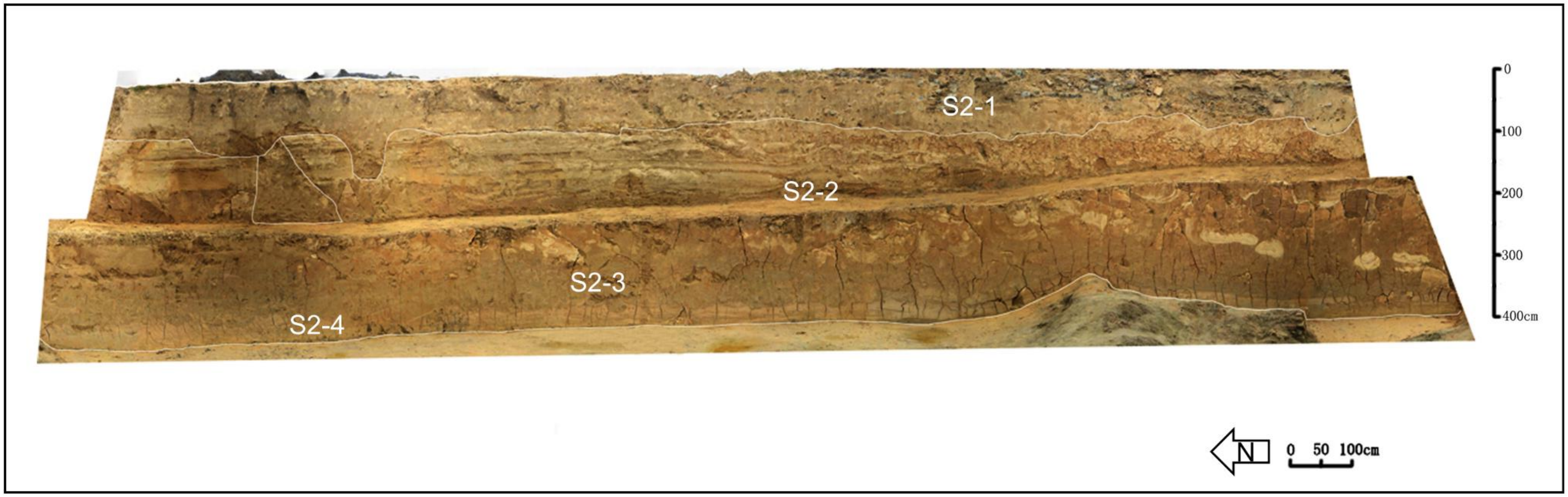

Supplementary Figure 10. Sedimentary profile at Yongning Wangfu.

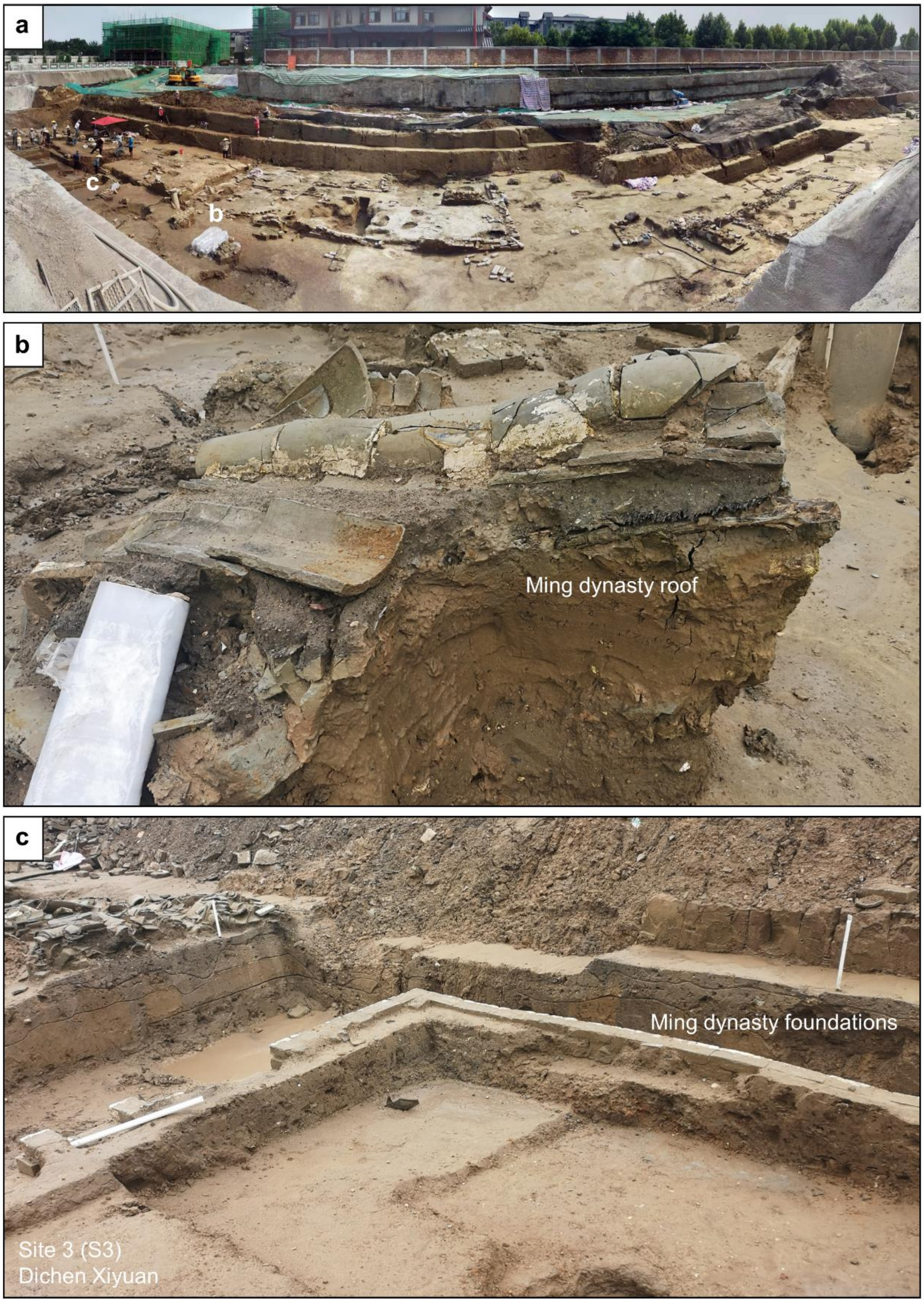

Supplementary Figure 11. Dichen Xiyuan. a, Panorama of Dichen Xiyuan with locations of photographs. b, a fallen roof with tiles and underlying matting still intact after the AD 1642 flood. c, an example of Ming dynasty building foundation buried by the AD 1642 flood.

Dynasty	Name	Location	Years capital
Xia<br>(c. 2070-1600 BC)	Lao Qiu	Guoduli Village<br>Kaifeng City	1849-1682 BC
Wei State<br>(c. 403-225)	Da Liang	Kaifeng, Northwest	364-225 BC
Later Liang<br>(AD 907-923)	Dongdu	Kaifeng City	AD 907-909<br>AD 913-923
Later Jin<br>(AD 936-947)	Dongjing	Kaifeng City	AD 938-946
Later Han<br>(AD 947-951)	Dongjing	Kaifeng City	AD 947-950
Later Zhou<br>(AD 951-960)	Dongjing	Kaifeng City	AD 951-960
Song<br>(AD 960-1279)	Dongjing	Kaifeng City	AD 960-1127
Wei Ji	Bianjing	Kaifeng City	AD 1132-1137
Jin<br>(AD 1115-1234)	Nanjing	Kaifeng City	AD 1161, 1214-1232
Han Song	Bianliang	Kaifeng City	AD 1358-1359
Ming<br>(AD 1368-1644)	Beijing	Kaifeng City	AD 1368-1378

Supplementary Table 1. List of ancient China's dynasties that had a capital in Kaifeng

Site	Latitude	Longitude	Type	Number	Figure
Xinzhengmen	114° 18' 15.70" E	34° 47' 42.38" N	Radiocarbon	Beta 500051<br>Beta 535781<br>Beta 535782<br>Beta 535783	Supplementary Fig. 3b<br>Supplementary Fig. 1, 2b<br>Supplementary Fig. 1, 2b<br>Supplementary Fig. 1, 2a
Yongning Wangfu	114° 20' 11.08" E	34° 47' 50.69" N	Radiocarbon and inscription	Beta 5335791<br>Beta 5335792<br>Beta 500052<br>Stelae	Fig. 4g<br>Fig. 4g<br>Fig. 4i<br>Fig. 4h
Dichen Xiyuan	114° 20' 30.51" E	34°48' 13.71" N	Radiocarbon	Beta 535785	Fig. 6c
Xinjiekou	114° 20' 40.31" E	34° 48' 0.19" N	Inscription	Artifact number JZ9: 308	Fig. 7a
Yulongwan	114° 21' 19.09" E	34° 47' 7.57" N	Radiocarbon	Beta 535789<br>Beta 535790<br>Artifact number: F3-8:664	Fig. 8c<br>No Fig.<br>No Fig.

Supplementary Table 2. Summary of excavated chronological evidence of the AD 1642 and AD 1842 Yellow River floods
